# Impact of COVID-19 Pandemic on Air Quality: A Systematic Review

**DOI:** 10.3390/ijerph19041950

**Published:** 2022-02-10

**Authors:** Ana Catarina T. Silva, Pedro T. B. S. Branco, Sofia I. V. Sousa

**Affiliations:** 1 LEPABE—Laboratory for Process Engineering, Environment, Biotechnology and Energy, Faculty of Engineering, University of Porto, Rua Dr. Roberto Frias, 4200-465 Porto, Portugal; up201605702@edu.fe.up.pt (A.C.T.S.); p.branco@fe.up.pt (P.T.B.S.B.); 2 ALiCE—Associate Laboratory in Chemical Engineering, Faculty of Engineering, University of Porto, Rua Dr. Roberto Frias, 4200-465 Porto, Portugal

**Keywords:** air quality, COVID-19, lockdown, air pollution, SARS-CoV-2

## Abstract

With the emergence of the COVID-19 pandemic, several governments imposed severe restrictions on socio-economic activities, putting most of the world population into a general lockdown in March 2020. Although scattered, studies on this topic worldwide have rapidly emerged in the literature. Hence, this systematic review aimed to identify and discuss the scientifically validated literature that evaluated the impact of the COVID-19 pandemic and associated restrictions on air quality. Thus, a total of 114 studies that quantified the impact of the COVID-19 pandemic on air quality through monitoring were selected from three databases. The most evaluated countries were India and China; all the studies intended to evaluate the impact of the pandemic on air quality, mainly concerning PM_10_, PM_2__.__5_, NO_2_, O_3_, CO, and SO_2_. Most of them focused on the 1st lockdown, comparing with the pre- and post-lockdown periods and usually in urban areas. Many studies conducted a descriptive analysis, while others complemented it with more advanced statistical analysis. Although using different methodologies, some studies reported a temporary air quality improvement during the lockdown. More studies are still needed, comparing different lockdown and lifting periods and, in other areas, for a definition of better-targeted policies to reduce air pollution.

## 1. Introduction

Air pollution was estimated to cause 4.2 million premature deaths worldwide in 2016, according to estimations made by the World Health Organization (WHO) [1], reaching almost 5 million in 2017 according to the Health Effects Institute [2]. Hence, this should be more than enough to motivate more aggressive policies to reduce air pollution. On 11 March 2020, the WHO declared the global pandemic of the novel coronavirus (COVID-19) [3]. Due to its transmissibility and rapid spread worldwide, several governments imposed severe restrictions on both social life and economic activity, including stay-at-home orders, social distancing, mandatory quarantines and remote work and school. Those restrictions put most of the global population into a general lockdown on late March 2020 (around 157 countries [4]), which temporarily reduced some of the major anthropogenic emission sources of air pollution and, consequently, resulted in marked air quality improvements worldwide [5]. Although those measures were temporary and possessed a high socioeconomic impact, they were both a global and local-scale unique opportunity to evaluate the effectiveness of some short-term measures to reduce air pollution in real-world. Thus, understanding the effects of the COVID-19 pandemic on air quality is a unique opportunity to define better targeted short and long-term policies to ameliorate the air quality, derived from the restrictions imposed by the governments.

Hence, it is important to conduct a detailed review of the studies that have been published so far about this topic, so that future policies based on the findings achieved can be proposed to improve air quality.

Some review papers (around 15) have been published concerning air quality improvement due to the lockdown effects [6,7,8,9,10], presenting general overviews. Besides, most of these review papers related the lockdown effect on air quality with other themes, such as water quality, noise pollution, energy consumption, and socioeconomic context. Moreover, as far as the authors’ knowledge goes, a systematic review has not yet been published.

Thus, the main aim of this study was to identify and discuss the scientifically validated literature that evaluated the impact of the COVID-19 pandemic and associated restrictions on air quality. Specifically, it intended to identify and discuss: (i) the impacts of the pandemic and associated restrictions on air pollutants’ concentrations; (ii) where the monitoring occurred in the reviewed studies; and (iii) the methodologies used for data analysis in different countries. This review only focused on studies that used air pollutants’ concentrations obtained through monitoring.

## 2. Materials and Methods

The present review includes studies published in the following databases: Science Direct, Scopus and PubMed. The keywords used in the first two databases were “Air Quality”, “Lockdown” and “Impact of COVID-19”, while, in PubMed, the keywords used were “Air Quality”, “Lockdown”, “Impact” and “COVID-19”. In the Scopus database, the search was limited to the subject area “Environmental Science”. No language restrictions were imposed during the search. Considering that COVID-19 was a recent phenomenon, there were no date restrictions imposed during the search. Therefore, all the studies fully published and in press until 27 April 2021 were considered.

A total of 439 articles were found with potential interest from the initial search. After removing duplicates, 266 articles were screened and their titles and abstracts appropriately reviewed. After this, articles were excluded based on the following criteria: (i) The study focused not only on the air quality, but also on other environmental compartments or themes, such as water quality, noise pollution, etc.; (ii) Air quality data was obtained only through modelling or satellite data; (iii) The study only considered an air quality index (AQI) instead of the air pollutants’ concentrations; (iv) The pollutants’ concentrations were not obtained from monitoring sites.

**Figure 1:**
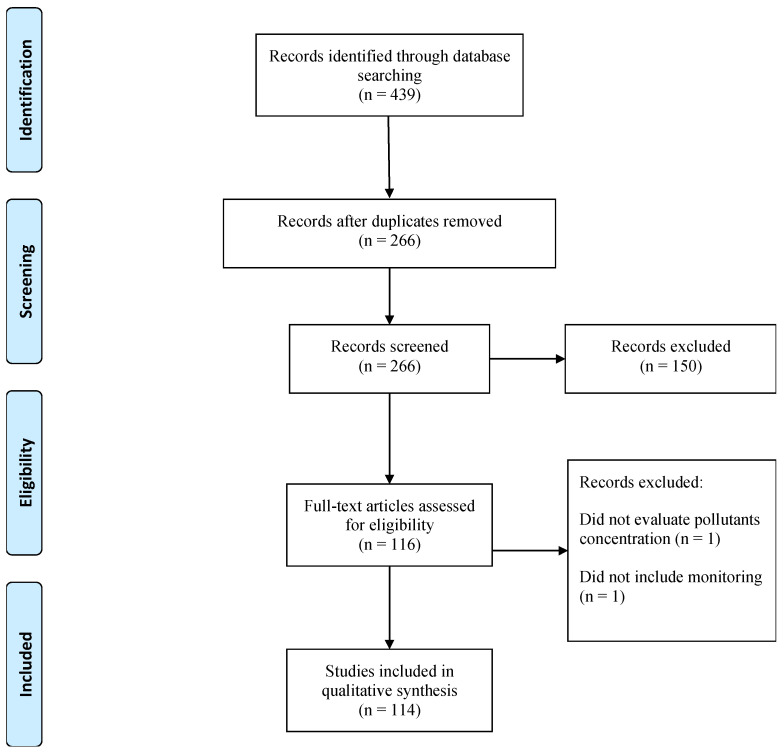
Systematic review flowchart (Adapted from Moher et al. [11]).

Hence, applying the exclusion criteria resulted in 114 articles. Figure 1 illustrates the flowchart with the numbers of studies identified, included and excluded.

## 3. Results

### 3.1. Study Design and Main Conclusions

Table S1 (in the Supplementary Material) summarises the information of the 114 articles reviewed, namely, study location, main objectives and data collected, methodology, and main conclusions. The summary of 30 studies that used as reference data an historical period of at least five years for comparison purposes, hence increasing the robustness of the analyses, are represented in Table 1. In this table, the main conclusions only refer to the pollutants’ behaviour, while, in the Supplementary Material, other withdrawn conclusions were included.

**Figure 2:**
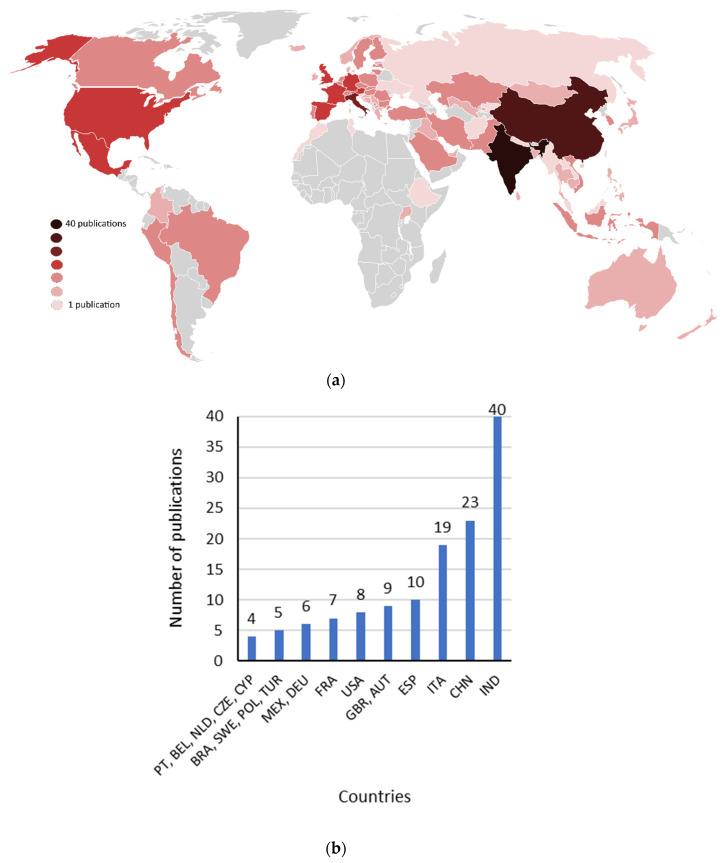
(**a**) Geographic representation on the world map of the studied locations from the 114 articles reviewed; (**b**) number of publications of the studied locations with at least 4 publications.

Figure 2 shows the study location of the 114 studies reviewed (Figure 2a) and the number of publications of the studied locations (Figure 2b). At least one study was developed in each continent. Ten publications reported studies conducted at a multinational level, namely: (i) Southeast Asia Region [40]; (ii) The United States of America and China [41]; (iii) worldwide [39,42,43]; (iv) America and Europe [44]; (v) Europe [12,45,46]; and (vi) The United States of America, India, China, and Europe [37]. India and China were the most studied countries, representing 55%, while 35% of the 114 publications were in India (e.g., [47,48]) From the 30 included in Table 1, it is possible to see more predominant studies from Europe (13 studies), but Asia was the focus of a similar number of studies (10).

The main objective of all the reviewed papers was to study and quantify the impact of the COVID-19 pandemic, namely the lockdown (considering different periods in some cases), on air quality.

However, some studies also intended to associate the impact of COVID-19 on air pollution with other variables. Some examples include energy consumption [48], general and/or COVID-19 mortality [49,50,51,52], other health impacts [24,27,53,54,55], traffic [17,23,44,56], and mobility trends [21,34,57].

**Figure 3:**
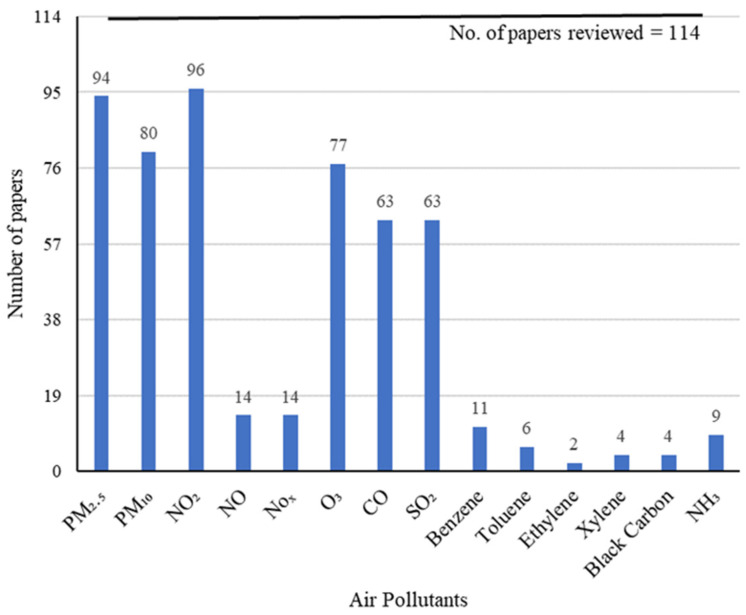
Graphical representation of the pollutants evaluated among the 114 articles reviewed.

Regarding data collected, and, specifically the evaluated pollutants, nitrogen dioxide (NO_2_) and suspended particles with an equivalent aerodynamic diameter smaller than 2.5 µm (PM_2__.__5_) and 10 µm (PM_10_) were the most analysed, followed by ozone (O_3_), carbon monoxide (CO), and sulphur dioxide (SO_2_) (Figure 3).

Other pollutants were also evaluated, namely non-methane hydrocarbons (NMHC) and total hydrocarbons (THC) [58]. Viteri et al. [22] also analysed NMHC, while Jain et al. [59] evaluated both suspended particles with an equivalent aerodynamic diameter smaller than 1 µm (PM_1_) and carbon dioxide (CO_2_); Dinoi et al. [16] investigated the influence of the COVID-19 pandemic on submicron particles.

Another very important factor when analysing air pollution data is the temporal resolution. This characteristic may impact the results, depending on the analysis performed. Most of the studies considered daily averages, although other temporal resolutions were also used, namely hourly data and monthly averages (more common), and weekly means, daily maximum of 8-h running means (especially for O_3_), daily maximum of 1-h average (for NO_2_), or even annual average. Considering an average of the whole study period was also a common practice observed in the reviewed studies (e.g., [52,60,61]).

In addition to the air pollutants’ concentrations, some studies considered other variables to complement the investigation developed. Those corresponded mainly to meteorological conditions, AQI and satellite data, representing 51%, 28% and 27%, respectively, of the total articles reviewed. As the weather conditions strongly influence air quality creating seasonal trends, more than half of the reviewed studies included meteorological variables (e.g., temperature, relative humidity, rainfall, wind speed and direction, solar radiation), whereas others included historical data to account for those trends. In addition, 41% of the reviewed articles integrated both weather conditions and historical data to achieve more accurate results.

### 3.2. Methodologies Used

Only 23 out of the 114 papers included rural and suburban areas concerning the areas of influence studied. It should be emphasised that 13 out of those 23 articles that included suburban and rural areas corresponded to European countries, possibly revealing a higher data availability in these areas in Europe than in other regions of the world, and/or demonstrating a more significant concern of studying these areas in Europe.

All the reviewed studies defined the first COVID-19 lockdown period as the main period of study, and the majority even included the periods immediately before and after it so that a comparison between the pollutants’ concentrations in these periods could be established. Many studies (45 out of 114) did not compare with previous years (before 2020), they just compared between the periods of measurement. Others chose to compare data from the lockdown period with the same period in 2019 (27 studies) or with a historical period of the 5 previous years or more (30 studies, Table 1). As described above, using at least five years as reference data, such as the ones presented in Table 1, enables the influence of the seasonal trends to be negligible, enhancing the robustness of the studies.

Regarding statistical analysis, 36 out of the 114 studies used simple descriptive statistical data analysis or a difference-in-difference analysis. Still, other studies went further in the statistical analysis and other more advanced statistical approaches were used, including: (i) significance testing (t-tests, Wilcoxon signed-rank test, Wilcoxon rank sum test, ANOVA, Kruskall–Wallis rank sum test, Duncan’s multiple range test, F-test, Dunn’s test); (ii) correlation analysis (Pearson, Spearman and Kendall rank correlation); (iii) cluster analysis (principal component analysis, hierarchical cluster analysis, and cluster analysis based on Euclidean distance and Ward’s methods); (iv) regression analysis (linear regression, including performance indexes such as coefficient of determination, root mean square error (RMSE), relative bias and mean absolute error (MBE), generalised linear model, generalised additive model, stepwise regression with backwards elimination, linear mixed effects model, Theil–Sen estimation, locally weighted scatterplot smoothing (LOWESS), simple ordinary least squares, functional concurrent regression model, Sen’s slope, breakpoint analysis and segment regression; (v) time-series (interrupted time-series analysis, time-series decomposition, Mann–Kendall test, multifractal time series analysis); (vi) geostatistical techniques (cokriging method, simple kriging, inverse distance weighting); (vii) probability distribution (generalised extreme value distribution); (viii) machine learning algorithms (random forest machine learning algorithm); and (ix) other methods, including panel regression, Fourier series and pollutants’ concentrations normalisation.

## 4. Discussion

The Asian region is one of the regions that suffers the most from air pollution; thus, it is natural that there would be a higher number of studies from this region. According to Rodríguez-Urrego [43], the capital cities from Pakistan, Iran, Kazakhstan, South Korea, and Singapore were included in the 50 most polluted cities of the world. Delhi is also often considered one of the most polluted cities. Moreover, PM and NO_2_, two of the most concerning air pollutants for human health, are largely emitted in those places [26,43], and have been covered by the studies published so far, which emphasises the relevance of quantifying the impact of the COVID-19 pandemic on air quality in the most polluted places.

Overall, the reviewed studies concluded that air quality improved during the lockdown compared with the pre-lockdown [62,63,64]. Some studies also reported increases for the post-lockdown periods because pollutants’ concentration increased to the pre-lockdown levels as soon as the lockdown period ended [65,66,67,68,69]. Nevertheless, not all evidenced an increase of the pollutants after the lockdown, because of the slow economic recovery [48,61,70]. Similarly, studies that compared to the same period in previous years [71,72,73,74,75,76,77,78,79,80,81,82,83,84,85,86,87], and even more robustly with historical data of more than 5 years (Table 1), reported a decrease of pollutants’ concentrations during the lockdown. As shown in Table 1, decreases between 9–60%, 21.4–61.6%, and 30–66% were obtained for PM_2__.__5_, PM_10_, and NO_2_ respectively (pollutants that were consistently reduced).The studies that also used satellite data corroborated the results obtained with the ground-based levels [33,57,88,89,90], and those that included AQI claimed that it improved during the lockdown period [54,91,92,93,94,95,96,97,98,99,100]. Besides, the higher levels of reduction were mainly found for the new industrialised areas, e.g., India and China [101]. 

The most studied pollutants were PM_2__.__5_, PM_10_, and NO_2_ since these are largely emitted especially by traffic in urban sites and, consequently, more strongly impact human health, particularly in Asian countries such as China and India, being also the most monitored. As for the remaining pollutants, they also significantly contribute to air pollution globally and, thus, are often found in the monitoring stations [42]. For example, Lian et al. [102] evaluated PM_2__.__5_, NO_2_, O_3_, PM_10_, CO, and SO_2_, once all of them are analysed by the State Control Station (China). Specifically, PM_2__.__5_, PM_10_ and NO_2_ consistently reduced in every part of the globe compared to the historical and the pre- and post-lockdown periods. Regarding the geographical distribution, the highest reductions were achieved in India and China, being mostly from 20% to 30% for PM and 30% to 60% for NO_2_, as expected given the high air pollution levels in those locations. The higher reductions were obtained when comparing the lockdown with the pre- and post-lockdown periods.

Concerning O_3_, an increase was evidenced in almost every study (e.g., [70,103]). Some authors correlated this O_3_ increment with the reduction of NO and the increase of solar radiation [104,105,106,107]. Specifically, Collivignarelli et al. [108] correlated the increase of this later pollutant with the high levels of benzene, noticed during the lockdown in Milan. Nevertheless, in some cities, O_3_ concentration was also reduced, mainly due to unfavourable weather conditions for this pollutant’s production [12,109,110,111,112,113]. Furthermore, Donzelli et al. [114] claimed that O_3_ was not monitored in urban/suburban sites; hence, significant conclusions about this pollutant’s behaviour were not drawn.

SO_2_ and CO results were not as consistent, having been more dependent on the location [28,56], presenting increases, decreases and, sometimes, remaining unchanged [42]. Part of the reviewed studies reported a decrease in CO concentrations, in some cases even higher than those of PM and NO_2_, e.g., in California (USA) [35]. Furthermore, a reduction of submicron particles was found during the lockdown [16].

Concerning the air pollution data temporal resolution, several temporal resolutions were chosen, nevertheless, and given the studies’ main objective, this factor apparently did not represent a major influence on the major conclusions.

Regarding the studies that used both weather conditions and a historical period as reference data, Marinello et al. [115] demonstrated that taking into account the meteorological conditions is very important, even when comparing to the previous year of the COVID-19 (2019) pandemic, since the weather conditions revealed an influence on the air pollutants’ dispersion and, consequently, on the improvement of the air quality, during the lockdown. Huang et al. [116] claimed a similar idea but highlighted the importance of the weather conditions in studies that compared periods of measurement in the same year.

Most of the authors investigated urban sites, not only because the lockdown restrictions more strongly impacted the cities rather than the rural and suburban locations, but also due to the higher contribution of urban areas for air pollution and the greater availability of air quality data there [36]. As a result, the air pollutant’s concentration, especially the traffic-related pollutants such as PM and NO_2_, reduced more in the urban areas, as described above. In addition, the studies that also evaluated other areas besides urban did not clearly discuss where the major impact was noticed, with the exception of Sannino et al. [117], who claimed a lower impact in the background areas due to the lockdown compared to urban traffic and industrial sites.

Although various statistical analyses were used, significance tests (t-tests) and correlation analysis were the most adopted besides descriptive analysis. A general reduction of the pollutants’ concentration was observed concerning the conclusions obtained from the studies. Hence, even though many different statistical analyses were performed, the similarity in the major findings reveals that comparing the conclusions achieved between the different studies is feasible and the results obtained are robust and credible. Yet, the most robust statistical analysis should be favoured, as they can give more credible quantifiable results. By using data collected in fixed monitoring stations, geostatistical techniques, such as inverse distance weighting and kriging, are robust methods to quantify populations’ exposure to air pollution [118]; they can serve as the basis for health impact assessment.

Some significant findings extracted from the reviewed studies that also intended to associate the impact of COVID-19 on air pollution with other variables were: (i) during the lockdown the energy consumption reduced; (ii) the reduction of air pollution led to a higher number of avoided premature-deaths, emphasising the health benefits from the air quality improvement achieved, as well as an avoided-economic cost; (iii) the traffic and public transport usage reduced during the lockdown period; (iv) the decrease in the number of vehicles circulating was one of the main sources that contributed to the air pollutant’s concentration reduction. The importance of creating policies that enable traffic emissions’ reduction was emphasized by Gao et al. [56]. In parallel with the main objective, Mehmood et al. [119] also intended to investigate the possible correlation between air pollutants and COVID-19, including the predicted number of infected cases, peak time, impact on the healthcare system and mortality. On the other hand, Dasgupta and Srikanth [120] qualitatively analysed the impact of COVID-19 restrictions on air quality in conjunction with city level socioeconomic parameters and policies to gain insights on the scope for integrating improved air quality with economic recovery for a sustainable transition. Vultaggio et al. [14] also evaluated the advantages of the lockdown as a measure to reduce air pollution, while Skirienė and Stasiškienė [45] investigated the association between industrial production index during the COVID-19 pandemic and air quality changes. Zhou et al. [31] associated the normalised difference vegetation index (NDVI) with air pollution during and after the lockdown. Other significant findings withdrawn from these reviewed studies were: (i) the air pollutants and COVID-19 revealed poor association (e.g.: [119]); (ii) the industrial production index was poorly correlated with the air quality changes (e.g.: [45]); (iii) a higher vegetation coverage induced a higher improvement on air quality (e.g.: [31]); (iv) the lockdown brought an opportunity to rethink new policies to ameliorate the air quality, considering a sustainable economic development (e.g.: [121,122,123,124,125,126,127]).

## 5. Conclusions

The present systematic review allowed us to summarise the information available in the literature that had been released about the impact of the COVID-19 pandemic on air quality.

The most evaluated countries consisted of those highly affected by air pollution (India and China), even though at least one study was conducted in every continent. Furthermore, the critical pollutants (PM_10_, PM_2__.__5_, NO_2_, O_3_, CO, SO_2_) were the most studied, particularly during the 1st lockdown in 2020 and mainly in urban areas, which are frequently more affected by air pollution. The pre- and post-lockdown periods were the periods most used for comparison, although comparisons with historical data (same period in previous years) also occurred. Several studies conducted a descriptive analysis, but many others complemented it with statistical analyses, which were diverse among the studies but led to similar conclusions. To have more credible quantifiable results, the most robust statistical analysis should be favoured, including geostatistical techniques that allow for estimating populations’ exposure and health impact assessment. Overall, similar findings were achieved among the studies, claiming a general improvement in the air quality during the lockdown compared to the pre-lockdown, post-lockdown (yet temporarily when compared with these periods), and historical periods. In particular, NO_2_ and PM were especially reduced in countries with higher air pollution levels; O_3_ registered mainly an increase, while SO_2_ and CO evidenced more diverse results.

Future work would benefit from: (i) the widening of the analysis concerning the study area, if possible, since little is known about the suburban and rural areas in other countries besides Europe (which was the continent that mainly evaluated these areas); (ii) the evaluation of the impact of the restrictions imposed during the pandemic beyond the 1st lockdown, and comparison of the effectiveness of those restrictions based on the 1st lockdown, since it was the period with the highest impact on citizens’ lives due to its novelty; (iii) the understanding of the impact of the lockdown on air quality under different meteorological conditions and the role that the weather conditions would play in the improvement or not of the air quality during the lockdown; (iv) the assessment of the impact of the COVID-19 pandemic on air quality attending the different social-economic sectors, i.e., tourism, services (such as public transportation, cafes, restaurants, etc.), industries, among others, and emission sources (e.g., residential); (v) the definition of better targeted and more effective policies to reduce air pollution both at a global and local scale; and (vi) to assess the health and economic burden avoided due to the air pollution reduction during the COVID-19 lockdown.

## Figures and Tables

**Table 1:** Summary of the main characteristics of the 30 reviewed studies (that used as reference data an historical period of at least 5 years), namely reference, location studied, main objectives, data, methodology, statistical analysis, and conclusions.

Reference	Location	Main Aim	Data			Methodology			Main Conclusions
			Main Pollutants	Temporal Resolution	Other Variables	Areas of Influence (Nº Monitoring Sites)	Period of Measurement	Statistical Analysis	
Europe									
[12]	Europe	Study the lockdown impact on NO_2_ and O_3_	NO_2_, O_3_	Daily max 1 h mean (NO_2_), daily max 8 h mean (O_3_)	T, wind components, Geopotential Height, Precipitation, 2-mspecific humidity, solar radiation	Urban background and rural (1331)	15 March to 30 April 2020	Generalised Additive Model	In 80% of sites studied NO_2_ decreased 5–55%, and O_3_ increased 5–22%, except in the Iberia Peninsula (lowered about 7%)
[13]	Lombardy, Italy	Assess the lockdown impact on air quality, using ground-level measurements and scenarios simulations with CAMx	NO_2_	Daily average	T, RH, WS, Precipitation	Urban traffic (5), and urban background (1)	2 periods in 2020: Pre-lockdown: 1 January to 7 March Lockdown: 8 March to 30 April	Kruskal—Wallis rank sum test, Mann-Whitney- Wilcoxon test	NO_2_ reduced 4.3– 33.7% based on the scenarios created, which was validated by the decreased registered with the monitoring sites data
[14]	Palermo, Italy	Assess changes on air quality due to the lockdown	CO, NO_2_, O_3_, PM_10_	Hourly mean, daily mean (only for PM_10_)	N/A	Urban Traffic (11)	1 January 1 to 31 July 2020	Two-tailed paired t-test	CO, NO_2_, and PM_10_ reduced around 51%, 50%, and 45% in the lockdown, whereas O_3_ increased
[15]	Vienna, Austria	Study the lockdown impact, namely road transportation changes, on air quality, and weather conditions influence	NO_2_, O_3_	Hourly	Total oxidant (O_x_), Monthly average daily traffic counts, mobility data (from Google and Apple), WS, WD, T, P, RH	Urban traffic, urban background, suburban background, suburban traffic and suburban industrial (17)	16 February to 30 September 2020 (lockdown—16 March to 13 April 2020)	Random forest machine learning algorithm, Mann-Whitney U-test	NO_2_ reduced around 13.7–30.4%, while O_3_ increased about 3.7–11.0%
[16]	Southern Italy	Study the impact of the lockdown on air quality, namely size and concentration of submicron particles	Submicron particles	Daily average	T, RH, Rainfall, WS, WD, size particles data	Urban background (1),—suburban (1)	3 periods in 2020: Pre- Lockdown: 1 January to 9 March Lockdown: 10 March to 17 May Post-Lockdown: 18 May to 31 July	Mann-Whitney U-test	Submicron particles reduced about 4% to 23%.
[17]	Portugal	Assess the impact of the lockdown on air quality	NO_2_, PM_10_	Hourly, daily average	Mobility data	Rural (9), urban background (14) and urban traffic (11)	2 periods, in 2020: Lockdown: 1 January to 15 March Lifting: 16 March to 31 May	Descriptive Statistics	- NO_2_ and PM_10_ diminished around 41% and 18%, with NO_2_ reduction above 60% on urban areas - Light increase on NO_2_ and PM_10_ concentration was noticed in the last 2 weeks of May
[18]	Po Valley, Italy	Study the effects of the lockdown, namely the anthropogenic emissions’ reduction, on air quality	NO_2_, Benzene, NH_3_	Monthly average, daily average	N/A	Monitoring sites selected for NO_2_ (218), Benzene (62), and NH_3_ (14) from Emilia-Romagna, Lombardia, Piemonte, and Veneto	January to June 2020	Kolmogorov-Smirnov test	- NO_2_ and benzene (traffic-related) decreased about 35–40% - NH_3_ (agriculture-related), did not significantly changed
[19]	Graz, Austria	Assess the influence of the lockdown on air quality	O_3_, PM_10_, NO_2_	Average concentrations	Traffic data, total oxidant (O_x_), T, RH, P, WS, WD, precipitation	Traffic, industrial, urban background (5)	January to May 2020	Principal Component Analysis, Random Forest Regression	PM_10_ and NO_2_ decreased during lockdown, whereas O_3_ increased
[20]	Italy	Assess the impact of the restrictive measures on air quality	PM_10_, PM_2__.__5_, NO_2_	Weekly average	N/A	Not specified	24 February to 4 May 2020	Panel regression	- PM_10_ and NO_2_ decreased about 5.125 µg/m^3^ and 5.375 µg/m^3^ - PM_2__.__5_ did not statistically significant changed
[21]	Turkey	Assess the impact of the lockdown on air quality in 81 cities from Turkey	PM_10_, SO_2_	Daily average	Mobility data, Car-purchasing data	Not specified (minimum of 81 sites)	January to November 2020	Welch’s t-test, F-test, Pearson’s correlation	- PM_10_ reduced 53.90 µg/m^3^- 43.75 µg/m^3^ during the lockdown - SO_2_ increased slighlty in the lockdown and significantly in the post-lockdown
[22]	Spain	Study the lockdown repercussion on air quality in 4 cities	SO_2_, CO, NO_2_, PM_10_, PM_2__.__5_, O_3_, BTXs, NH_3_	Monthly average	NMHC	Urban traffic (1), suburban background (1), industrial and residential influence (1), and national coverage background	2 periods, in 2020: Pre-Lockdown: January to February Lockdown and de-escalation: 14 March to 30 June	Student’s t-test, Mann-Whitney U test	NO_x_, BTX_s_, CO, NMHC, and NH_3′_ reduced statistically significant in March and April PM_10_ and PM_2__.__5_ changes were small due to natural and residential sources
Asia									
[23]	Almaty, Kazakhstan	Assess the changes on air quality, before and during the lockdown	PM_2__.__5_, BTEX, NO_2_, O_3_, SO_2_, CO	Daily and average concentrations, and 12-h average (BTEX)	WS, WD, T, RH, Precipitation	Road traffic; PM_2__.__5_: (7); BTEX: (6); NO_2_, O_3_, SO_2_, and CO (1)	PM_2__.__5_: Pre-lockdown: 21 February to 18 March Lockdown: 19 March to 14 April 2020 BTEX: Since end of March until beginning of April (3rd) Remaining: 2 March to 14 April 2020	Cokriging method	PM_2__.__5_, CO, and NO_2_, reduced about 21%, 49%, and 35%, while SO_2_ and O_3_ increased 7% (not statistically significant) and 15% (due to high insolation) High levels of benzene and toluene (101 µg/m^3^ and 67 µg/m^3^) due to coal-related sources (e.g: householding, power plants)
[24]	India	Study the impact of the lockdown and associated anthropogenic activities interruption on PM_2__.__5_ and aerosols, in 5 cities	PM_2__.__5_	Hourly average	AOD (satellite imagery)	Not specified	25 March to 11 May 2020	Generalised Extreme Value distribution	PM_2__.__5_ decreased from 10% to 52% in the total of the 5 cities
[25]	China	Study the impact of the lockdown on air quality	O_3_, NO_2_, CO, PM_2__.__5_, PM_10_, SO_2_	Average concentration	N/A	Not specified (1640)	January to April 2020, corresponding to the lockdown period from 23 January to 31 March 2020	Theil-Sen estimation, Locally Weighted Scatterplot Smoothing (LOWESS)	NO_2_, PM_2__.__5_, PM_10_ and CO decreased 27%, 10.5%, 21.4% and 12.1%, while O_3_ showed few changes
[26]	India	Study the influence of the lockdown on air quality in Delhi, Ahmedabad, Mumbai, and Pune	PM_2__.__5_, PM_10,_ NO_2_	Daily average	Rainfall, T	City coverage (32–40)	20 March to 15 April 2020	Descriptive Statistics	Overall, NO_2_, PM_2__.__5_, PM_10_ reduced 60–66%, 25–50%, and 46–50%
[27]	China	Study the impact of the lockdown on PM_2__.__5_	PM_2__.__5_	Daily average	Air pressure, total column water, wind components, T, total column ozone, RH and planetary boundary layer height, population, and mortality data	Not specified (1388)	Lockdown: February to March, 2020	Kolmogorov-Zurbenko filter and multiple linear regression	PM_2__.__5_ average concentrations decreased around 30–60%, with the national average concentrations reducing by 18 µg/m^3^
[28]	China	Evaluate the impact of the lockdown on air quality in Wuhan, Hubei, and China (excluding Hubei)	PM_2__.__5_,PM_10_, SO_2_, NO_2_, O_3_, CO	Daily average	N/A	Not specified (365)	21 January to 23 March 2020	Descriptive Statistics	NO_2_ reduced 53%, 50% and 30%, in Wuhan, Hubei and China, as well as PM_2__.__5_ by 35%, 29% and 19%, when compared to 2019 PM_10_ had similar reduction to PM_2__.__5_ SO_2_ and CO reduced but not as much as the before-mentioned pollutants O_3_ increased up to 58%, in Wuhan
[29]	National Capital Regional, India	Assess the impact of the lockdown on air quality	PM_10_, PM_2__.__5_ NO_x_, NO, NO_2_, NH_3_, SO_2_, CO, Benzene, O_3_	24-h average	RH, T, WS, solar radiation, AQI (calculated)	Monitoring sites from Delhi (20), Gurugram (4), Faridabad (4), Ghaziabad (4), and Noida (4)	1 March to 1 May 2020, with the lockdown on 25 March to 1 April	Pearson’s correlation, ANOVA	PM_10_, PM_2__.__5_, NO_x_, NO, NO_2_, SO_2_, CO, NH_3_ and Benzene reduced around 61.6%, 60.0%, 58.6%, 62.3%, 46.8%, 33%, 44.8%, 26.6% and 53%
[30]	Northern China	Study the impact of the lockdown on air quality, with minimization of weather and other environmental influences	PM_2__.__5_, NO_2_	Daily average	RH, WD, WS, Sea Level Pressure, planetary Boundary Layer Height	Not Specified	January to December 2020	Descriptive Statistics	PM_2__.__5_ and NO_2_ decreased 0.03 µg/m^3^ and 17.13 µg/m^3^
[31]	China	Evaluate the impact of the lockdown on air quality in 341 cities	NO_2_, CO, O_3_, PM_10_, PM_2__.__5_, SO_2_	Daily average, monthly average, 1-h, and 8-h (only for O_3_) average	AQI and Normalised Difference Vegetation Index (NDVI)	Not specified	1 January–31 June 2020, with the lockdown on 23 January to 27 March	Pearson’s correlation, t-test, linear regression	Overall, comparing pre- and during the lockdown periods, PM_2__.__5_, PM_10_, SO_2_, CO and NO_2_ reduced by 35.59%, 38.52%, 20.81%, 31.10% and 55.10%, and O_3_ increased by 82.52% This behaviour was also observed when comparing the data with previous years
America									
[32]	Sommerville, USA	Study the changes on air quality due to traffic-reduction, due to the lockdown	Black Carbon, PM_2__.__5_, NO_2_	Daily	Total Traffic Volume, T, WS	Traffic, near I-93 route (1) and urban background (1)	24 March–15 May 2020	Wilcoxon Rank Sum test	Black carbon reduced 51% (both sites), NO_2_ reduced 30% (traffic) and 47% (urban background), and PM_2__.__5_ lowered 9% (traffic—near I-93 roadway) and 52% (urban background)
[33]	São Paulo, Brazil	Study the effects on air quality, due to the partial lockdown	PM_10_, PM_2__.__5_, CO, NO, NO_2_, NO_x_, SO_2_, O_3_	Monthly average	NO_2_ (satellite data)	Urban traffic (2), urban industrial (1) and influence on a city centre (1)	2 periods in 2020: Before partial-lockdown: 25 February to 23 March Partial-lockdown: 24 March to 20 April	Descriptive Statistics	NO, NO_2_, CO, and PM_2__.__5_ reduced by 48.6–77.3%, 30.1–54.3%, and 36.1–64.8%, and 29.8%, while O_3_ increased by 30%
[34]	Mexico	Study the impact of the the lockdownon air quality	SO_2_, NO_2_, CO, PM_10_, PM_2__.__5_, O_3_	Average concentration	Average traffic count, T, RH, WS, Precipitation	Not specified	2 periods in 2020: Pre-lockdown: 1 January to 31 March Lockdown: 1st phase: 1–30 April 2nd phase: 1–31 May	Correlation tests	Compared to the pre-lockdown period, SO_2_, NO_2_ and PM_10_ reduced by 55%, 29% and 11%, whereas O_3_, CO and PM_2__.__5_ increased around 63%, 1.1% and 19%, respectively In comparison to the 2015–2019, NO_2_, SO_2_, CO, PM_10_ and PM_2__.__5_ reduced by 19–36%, and O_3_ decreased around 14%
[35]	California, USA	Assess the changes on air quality due to the lockdown	NO_2,_ O_3,_ PM_2__.__5_, PM_10,_ CO	Daily average	NO_2_ (satellite data), main power plants, highways, and wildfire’s location	Not Specified	3 periods in 2020 Pre-lockdown: 26 January to 18 March Lockdown: 19 March to 8 May Post-lockdown: 9 May to 14 June	Pollutants’ concentrations Normalization	CO reduced more than NO_2_ and PM_2__.__5_ during lockdown NO_2_ increased in residential and transportation hub areas.
Oceania									
[36]	Auckland, New Zealand	Study the impact of the lockdown on air quality	PM_10_, PM_2__.__5_, Black Carbon, O_3_, NO_2_	24-h average	NO_2_ (satellite data), T, RH, WS, Rainfall, traffic data	Urban (1), suburban roadside (1), and urban background (1)	February to April 2020, being the lockdown during 27 March until 17 April	t-tests	The pollutants reduced, except O_3_ which increased Black carbon and NO_2_ reduced the most
Multi-country									
[37]	USA, India, China, and Europe	Assess the impact of the measures implemented on a multi-scale, on air quality	O_3_, PM_2__.__5_, SO_2_, CO, PM_10_, NO_2_	Monthly average	NO_2_ (satellite data)	Not specified	January to April, 2020	Statistical approach developed by [38]	The pollutants reduced, except O_3_ which increased In some European cities, besides O_3_ other pollutants increased contrarily to other countries—In New Delhi O_3_ did not increase
[39]	Worldwide	Investigate the impact of the lockdown on air quality	PM_2__.__5_, NO_2_, O_3_	Daily average, monthly average	N/A	Urban and/only traffic, background, industrial, semi-rural area (458)	1 January to 30 April 2020	Signed Rank test, Paired t-test, ANOVA, Time Series Decomposition	NO_2_ and O_3_ had the reduction and increase globally, respectively. PM_2__.__5_ also reduced globally

max—Maximum; h—Hour; T—Air Temperature; CAMx—Comprehensive Air Quality Model with Extension; RH—Relative Humidity; WS—Wind Speed; N/A—Not Applicable; WD—Wind Direction; P- Precipitation; NMHC—Non-Methane Hydrocarbons; AOD—Aerosol Optical Depth; AQI—Air Quality Index; ANOVA—Analysis of Variance; WHO—World Health Organization; NDVI—Normalised Difference Vegetation Index.

## Data Availability

Not applicable.

## Supplementary Materials

The following supporting information can be downloaded at: https://www.mdpi.com/article/10.3390/ijerph19041950/s1, Table S1: Summary of the main characteristics of 114 reviewed studies, namely reference, location studied, main objectives, data, methodology, statistical analysis, and conclusions.
