# Supplementary material for: Impact of COVID-19 Pandemic on Air Quality: A Systematic Review
Source: Int J Environ Res Public Health. 2022 Feb 10;19(4):1950. doi: 10.3390/ijerph19041950 (PMC8871899; doi:10.3390/ijerph19041950)
Supplement: Supplementary file 1 [file ijerph-19-01950-s001.zip › ijerph-1562180-supplementary.pdf]

Table S1. Summary of the main characteristics of 114 reviewed studies, namely reference, location studied, main objectives, data, methodology, statistical analysis, and conclusions.

			Data			Methodology				Main conclusions	
Reference	Location	Main Aim	Main Pollutants	Temporal resolution	Other Variables	Areas of Influence (Nº monitoring sites)	Period of Measurement	Reference Data	Statistical Analysis	Pollutants' Behaviour	Other conclusions
[53]	Shanghai Municipality, China	Assess the impact on air quality due to lockdown in 16 districts of Shanghai	$PM_{2.5}$, SO_2, NO_2, CO, PM_{10}, O_3	Daily average	AQI	Downtown and suburban	3 periods:<br>- Pre-Lockdown: November 25th, 2019 to January 23rd, 2020<br>- Lockdown: January 24th to March 23rd, 2020<br>- Post-Lockdown: March 24th to May 22nd, 2020	Comparison between periods of measurement and the same period in 2019, starting in November 2018, as well as between downtown and suburban areas, in each period	Wilcoxon Signed-Rank Test	- During the lockdown, relatively to the pre-lockdown, all pollutants' concentrations decreased, except O_3 (that increased)<br>- In comparison to 2018-2019, a significant reduction of air pollution was observed in the lockdown<br>- In the beginning of the lifting, the pollutants' concentrations raised	- The reduction of the pollutants was possibly due to traffic and industrial activities' interruption<br>- Lockdown had little impact on O_3. Its behaviour was possibly due to the meteorological conditions and NO_x decrease

[88]	Iran	Evaluate the impact of the lockdown on air quality in Iran, particularly in Tehran	NO_2, CO, O_3, PM_{10}, $PM_{2.5}$, SO_2	Average concentration	SO_2, NO_2, AOD (satellite data), meteorological conditions	Monitoring sites from Tehran (12)	2 periods, in 2020:<br><br>- Pre-Lockdown: February 20th to March 20th<br><br>- Lockdown: March 21st to April 21st	Same periods in 2019	Descriptive Statistics	SO_2, NO_2, CO and PM_{10} decreased by 5%–28%, 1%–33%, 5%–41%, and 1.4%–30%, respectively, whereas O_3 and $PM_{2.5}$ increased around 0.5%-103% and 2%-50%, respectively, during the lockdown, in comparison to the 2019 data in Tehran	- SO_2 and NO_2 concentrations, from satellite data, decreased during the lockdown, while AOD increased<br><br>- Further research is needed to understand the total effect of the weather conditions on air pollution
[65]	USA	Assess the impact of the 1st phase of the lockdown on air pollution	NO_2, CO, $PM_{2.5}$, PM_{10}, O_3	Not specified	N/A	Monitoring sites near metropolitan areas in 23 states (28)	March 15th to April 25th, 2020 (P1)	Pre-lockdown (P0): January 25th to March 7th, 2020 and with P1/P0 in 2017-2019	Spearman correlation	- NO_2 reduced from 5% to 49%, but only a decrease above 19% was considered to be statistically significant<br><br>- CO decreased significantly, while $PM_{2.5}$ and PM_{10} registered a non-uniform behaviour	The 1st phase of the lockdown affected more the air quality on urban areas than the suburban ones, even though temporarily

									- O_3 concentrations increased/decreased, depending on the state in question	
[108]	Metropolitan City of Milan (MeCIMI), Italy	Investigate the impact of the lockdown on air quality, in MeCIMI	PM_{10}, $PM_{2.5}$, Black Carbon, Benzene, CO, SO_2, NO_2, NO_x, O_3, NH_3	Daily average	Data of estimation of emission sources, WS, T, RH, Rainfall, Solar irradiance	Monitoring sites from Milan (5), SaA (6), and SaB (7)	3 periods in 2020:<br>- CTRL: February 7th to 20th<br>- Partial Lockdown: March 9th - 22nd<br>- Total Lockdown: March 23rd to April 5th	Comparison between the CTRL with partial and total lockdown periods	Descriptive Statistics	- CO registered the most significant reduction<br><br>- SO_2 decreased only in Milan city (monitoring site)<br><br>- O_3 increased during the total lockdown	O_3 production might have been influenced by the high benzene's concentrations in Milan
[114]	Italy	Study the changes on air quality due to lockdown in Florence, Pisa, and Lucca	PM_{10}, $PM_{2.5}$, NO_2, O_3	Daily average	Rainfall, WS, RH, T, Solar irradiance	Urban background (3), urban traffic (4), suburban background (1), and rural background (1)	3 periods in 2020:<br><br>- Pre-lockdown: January 1st to March 8th<br>- Lockdown: March 9th to June 3rd<br>- Post-lockdown:	Same period in 2019	Paired t-test	Overall, $PM_{2.5}$ and PM_{10} levels decreased insignificantly during the lockdown, whereas NO_2 reduced significantly	O_3 was only monitored in urban/suburban background monitoring sites, therefore significant conclusions could not be taken

							June 4th to August 12th				
[66]	Delhi, India	Assess the impact of the lockdown on air pollution	NO_x, O_3, $PM_{2.5}$, PM_{10}	Monthly average	N/A	Not specified (5)	2 Periods in 2020:<br><br>- Pre-Lockdown: February to March 23rd<br><br>- Lockdown: April	Reflected period in 2018-2019	Descriptive Statistics	- $PM_{2.5}$, PM_{10} and NO_x decreased about 50%, 68% and 70%, respectively<br><br>- O_3 reduced in the majority	It is expectable an increase of the pollutant's concentration, as soon as the lockdown period ends
[78]	Italy	Assess the changes on air quality due to the lockdown and to determine the contribution of the traffic-emissions in urban areas from 6 Italian cities	NO_2, O_3, $PM_{2.5}$, PM_{10}	Daily	Road Mobility, T, RH, WS, WD, Rainfall, RAD	Urban traffic, urban background, and suburban background	February 24th to April 30th, 2020	February 25th to May 2nd, 2019	Descriptive Statistics	- NO_2 decreased significantly<br><br>- $PM_{2.5}$ and PM_{10} registered minor reductions<br><br>- O_3 concentrations increased	Traffic emissions reduced 48%-60 %, in urban areas, and up to 80.1% on the entire motorway network

[93]	China	Study the effects of the lockdown on air quality	$PM_{2.5}$	Not specified	AQI, city-level lockdown policies	Not specified (1600)	January 1st to March 1st 2020	Comparison between the regions where the lockdown was and wasn't implemented	Descriptive Statistics	$PM_{2.5}$ showed a reduction of about 24% in regions with lockdown, when comparing to the non-lockdown imposed areas	- AQI reduced about 22%, in regions with lockdown, when comparing to the non-lockdown imposed areas<br><br>- These results proved to be statistically not significant<br><br>- The lockdown isn't a sustainable policy to ensure long-term improvement on air quality
[116]	Hong Kong, China	Understand the effect of the social restriction measures, including the	O_3, NO_2, CO, $PM_{2.5}$, PM_{10}, SO_2	Average concentrations	N/A	Traffic and residential (13)	2 periods in 2020:<br><br>- Pre-lockdown: January 1st-31st	Same period in 2017-2019	Descriptive Statistics	- From January to April, O_3 increased, whereas the remaining pollutants' concentrations decreased, since the road traffic highly diminished as well	Seasonal variables strongly influenced air quality, and not taking them into account drives

		lockdown, on the roadside air quality					- During lockdown: February to April 30th			- Comparing pre-COVID-19 period with the same in previous years, CO, SO_2, NO_2, PM_{10} and $PM_{2.5}$ reduced by 2%-17%, 17%-32%, 13%-18%, 14%-31%, and 19%-33%, respectively. During it, these pollutants diminished by 21%-40%, 42%-52%, 10%-28%, 8%-37%, and 1%-44%, respectively<br><br>- O_3, in pre- COVID-19 period, increased around 24%-39%, and 1%-72% during it, compared to the 2017-2019 reference period	to misleading conclusions, when comparing only the period pre-COVID-19 with the period during COVID-19 in the same year
[32]	Sommerville, USA	Study the changes on air quality due to	Black Carbon, $PM_{2.5}$, NO_2	Daily	Total Traffic Volume, T, WS	Traffic, near I-93 route (1) and urban	March 24th - May 15th 2020	Same period in 2015-2019	Wilcox on Rank Sum test	Black carbon reduced 51% (both sites), NO_2 reduced 30% (traffic) and 47% (urban background), and	Traffic reduced around 50% during lockdown

		traffic-reduction, due to the lockdown				background (1)				$PM_{2.5}$ lowered 9% (traffic - near I-93 roadway) and 52% (urban background)	
[121]	India	Assess the effect of the lockdown on air quality, in Delhi, Mumbai, Chennai, Bangalore, and Kolkata	NO_2, CO, O_3, PM_{10}, $PM_{2.5}$	Daily average	T, WS, RH	Monitoring sites, as city coverage, from Delhi (38), Mumbai (10), Chennai (4), Bangalore (10), and Kolkata (7)	2 periods in 2020:<br><br>- Pre-Lockdown: March 10th-20th<br><br>- Lockdown: March 25th to April 6th	Comparison between lockdown months in 2020, with March 20th to April 10th, 2019, and between pre- and during the lockdown period (2020)	Paired t-test, Kringing	- Overall, the pollutants' concentration reduced, whereas O_3 increased<br><br>- In Delhi, $PM_{2.5}$, PM_{10}, NO_2 and CO diminished around 41%, 52%, 50% and 29%, respectively, and O_3 increased by 7%, during the lockdown. In the same place, when compared to the 2019 data, $PM_{2.5}$, PM_{10}, NO_2, CO and O_3 registered a decrease by 32%, 34%, 27%, 27% and 11%<br><br>- Chennai demonstrated a similar reduction in PM_{10}, NO_2 and CO, when compared to	Similar pollutants' concentrations decline was obtained in Kolkata

									2019 data, whereas, between pre- and during the lockdown periods, $PM_{2.5}$, NO_2 and CO declined about 14%, 30% and 25%, with an O_3 increase by 3%	
[23]	Almaty, Kazakhstan	Assess the changes on air quality, before and during the lockdown	$PM_{2.5}$, BTEX, NO_2, O_3, SO_2, CO	Daily and average concentrations, and 12-h average (BTEX)	WS, WD, T, RH, Precipitation	Road traffic; $PM_{2.5}$: (7); BTEX: (6); NO_2, O_3, SO_2, and CO (1)	$PM_{2.5}$:<br>- Pre-lockdown: February 21st to March 18th<br>- Lockdown: March 19th to April 14th 2020<br><br>BTEX:<br>- Since end of March until beginning of April (3rd)<br><br>Remaining:	Reflected period in 2018 - 2019, for $PM_{2.5}$, 2015-2019 for BTEX, and comparison between pre- and during the lockdown periods	Cokriging method	- $PM_{2.5}$, CO, and NO_2, reduced about 21%, 49%, and 35%, while SO_2 and O_3 increased 7% (not statistically significant) and 15% (due to high insolation)<br>- High levels of benzene and toluene (101 μg/m^3 and 67 μg/m^3) due to coal-related sources (e.g: householding, power plants)	- $PM_{2.5}$ exceeded WHO guidelines for 18/27 days of the lockdown<br>- Significant decrease of CO and NO_2 occurred as a response to the traffic interruption<br><br>- The lockdown was not enough to produce a significant impact on air quality, due to others

							- March 2nd to April 14th 2020				emission sources many
[50]	Morocco	Determine if the lockdown had a positive effect on the air quality	NO_2, $PM_{2.5}$, CO	Daily	Air quality related to mortality (calculated), COVID-19 cases and deaths, population data	Not specified	2 periods in 2020:<br>- Pre-lockdown: February 16th to March 19th<br>- Lockdown: March 20th to April 20th	Same period in 2016-2019 and comparison between periods of measurement	Theil-Sen estimation, Mann-Kendall Test	During lockdown:<br>- NO_2 reduced 7 $\mu g/m^3$ to 12 $\mu g/m^3$<br>- CO diminished 0.04 mg/m^3 to 0.12 mg/m^3<br>-$PM_{2.5}$ reduced 14 $\mu g/m^3$ to 18 $\mu g/m^3$	- The air quality improved during the lockdown<br>- The reduction confirmed between the pre-lockdown and during the lockdown, was statistically significant, while in comparison with the 2016-2019 period it was not statistically significant<br>- In total, the avoided NO_2 related deaths were 215, and $PM_{2.5}$ related avoided deaths

											were 63, being the avoided deaths by cardiovascular diseases more than 60% of those
[24]	India	Study the impact of the lockdown and associated anthropogenic activities interruption on $PM_{2.5}$ and aerosols, in 5 cities	$PM_{2.5}$	Hourly average	AOD (satellite imagery)	Not specified	March 25th to May 11th 2020	Same period in 2015-2019	Generalised Extreme Value distribution	$PM_{2.5}$ decreased from 10% to 52% in the total of the 5 cities	- Other sources like industrial and commercial proved to be significantly contributors to the levels of $PM_{2.5}$<br>- AOD reduced 1%-29%, in the North, East, and South, increasing in central India.<br>- $PM_{2.5}$ reduction led to less 630 premature deaths and avoided-economic cost of 0.69 billion USD

[47]	India	Study the effect of the 1st phase of the lockdown on air quality in Delhi, Mumbai and Singrauli	NO_2, O_3, PM_{10}, $PM_{2.5}$, SO_2	Daily average, daily max 8h average (only for O_3)	NO_2 (satellite data)	Monitoring sites from Delhi (4), Mumbai (4) and Singrauli (1)	2 periods, in 2020:<br><br>- Pre-Lockdown: March 1st-24th<br><br>- 1st phase of the Lockdown: March 25th to April 15th	Comparison between periods of measurement	Descriptive Statistics	- Overall, PM_{10} and $PM_{2.5}$ concentrations diminished by 55.01% and 49.34%, during the lockdown, compared to the pre-lockdown period<br><br>- SO_2 in Delhi reduced by 19.51%, in comparison with the 2020 periods<br><br>- During the lockdown, $PM_{2.5}$, PM_{10}, and NO_2 registered a significant decrease in Delhi and Mumbai<br><br>- Overall, O_3 increased by 37.35%	- NO_2 concentrations from satellite data decreased around 65% to 75% in Delhi and Mumbai, compared to the pre-lockdown period. In relation to 2019, those reductions were about 40%-50%<br><br>- Air quality improved during the lockdown, compared to the pre-lockdown period and to 2019
[42]	World wide	Assess and quantify the	$PM_{2.5}$, PM_{10}, O_3, NO_2, SO_2	Monthly, daily average	Lockdown and lifting	Not specified (162)	January 1st to June 10th 2020	March, April and May 2019, and comparison	Descriptive Statistics	- In the more polluted cities (Beijing, Bengaluru, Delhi, Lima, Mumbai,	-Globally, $PM_{2.5}$, PM_{10} and NO_2 reduced in

		impact of the contigency measures on air quality in 12 cities highly affected by this phenomenon			dates of each city			between the pre-lockdown, during lockdown and post-lockdown		Rome, and Wuhan) $PM_{2.5}$ concentrations dropped around 20.2%-34.3%, PM_{10} decreased by 23.7%-47.3%, NO_2 diminished between 31.6% and 64.5% (highest reduction observed), in relation to 2019<br><br>- SO_2 revealed a mixed behaviour and O_3 increased during the lockdown	2020 in relation to 2019, and also diminished after the lockdown<br><br>- In the lifting, the pollutants' levels raised, therefore the improvement on air quality during the lockdown was temporary
[105]	India	Evaluate the changes on air quality due to the lockdown in 39 Indian cities	NO_2, CO, O_3, PM_{10}, $PM_{2.5}$, SO_2	Average concentration	NO_2 (satellite data), AQI	Not specified (39)	For Ghaziabad and Patiala:<br>- Pre-lockdown: February 1st to March 23rd, 2020<br><br>- Lockdown: March 24th	For Ghaziabad and Patiala:<br>- Same period in 2019 and comparison between periods of measurement	t-test	- In general, PM_{10}, $PM_{2.5}$, CO, NO_2 and SO_2 concentrations reduced up to 44%, 38%, 28%, 42% and 40%, respectively, during the lockdown<br><br>- O_3 manifested both increase and decrease trends, depending on the locations in matter	- The increase of O_3 was mainly due to NO concentration decrease<br><br>- Lockdown demonstrated an improvement on air quality. Hence, in

							to May 31st, 2020<br><br>For the remaining 37 cities:<br>- Lockdown: March 24th to May 31st, 2020	For the other cities:<br>- Same period in 2019			future scenarios of several air pollution, similar restrictions may be implemented
[57]	Singapore	Study and quantify the impact of the lockdown on air quality	NO_2, O_3, PM_{10}, $PM_{2.5}$, SO_2	Average concentration	AOD, NO_2, SO_2 (satellite data), T, RH, Precipitation, wind conditions, PSI, mobility data	Monitoring sites from North, East, West, Central and South of Singapore (5)	- Lockdown: April 7th to May 11th, 2020	Same period in 2016-2019	Wilcoxon signed-rank test, Spearman's rank correlation	Overall, PM_{10}, $PM_{2.5}$, NO_2, CO, and SO_2 concentration decreased by 23%, 29%, 54%, 6%, and 52%, respectively, whereas O_3 increased around 18%	- The reduction on NO_2 and SO_2 was corroborated by the satellite data, whose findings registered a decrease of both pollutants as well as AOD<br><br>- $PM_{2.5}$ and NO_2 were strongly correlated to the reduction of the mobility trend

											- PSI reduced by 19%
[95]	Nation al Capita l Region , India	Study the improve ment on air quality during the lockdown , and after it, in the ten most polluted cities from the National Capital Region, India	PM_{10}, $PM_{2.5}$, NO_2, SO_2, O_3, NO, CO, NH_3	Daily average, hourly (PM_{10} and $PM_{2.5}$)	AOD (satellite data), NAQI	Not Specified (59)	2 periods in 2020:<br>- Pre-lockdown: March 10th-20th<br>- Lockdown: March 21st to April 20th	Between periods of measuremen t	Descri ptive Statisti cs	- Overall, PM_{10} and $PM_{2.5}$ diminished until 49% and 46.9% respectively during the third week of the lockdown; NO_2 and CO reduced by 57% and 30% respectively during the total lockdown<br>- Little changes were observed for SO_2, and a slight increase in O_3 concentrations was registered	NAQI levels demonstrated that up to 70% of the Indian cities studied registered an improvement on air quality, achieving "satisfactory" and/or "good" categories
[33]	São Paulo, Brazil	Study the effects on air quality, due to the partial lockdown	PM_{10}, $PM_{2.5}$, CO, NO, NO_2, NO_x, SO_2, O_3	Monthly average	NO_2 (satellite data)	Urban traffic (2), urban industrial (1) and influence on a city centre (1)	2 periods in 2020:<br>-Before partial-lockdown: February 25th to March 23rd	Same period in 2015-2019	Descri ptive Statisti cs	NO, NO_2, CO, and $PM_{2.5}$ reduced by 48.6%- 77.3%, 30.1%- 54.3%, and 36.1% - 64.8%, and 29.8%, while<br>O_3 increased by 30%	- In the industrial area air pollution increased comparing the before and during partial lockdown<br>- It was noted that industries did not shut

							- Partial-lockdown: March 24th to April 20th				down during the partial lockdown<br>- NO_2 from satellite data decreased 45% and 27% relatively to 2019 and to the period before the partial-lockdown
[12]	Europe	Study the lockdown impact on NO_2 and O_3	NO_2, O_3	Daily max 1h average (NO_2), daily max 8h average (O_3)	T, wind components, Geopotential Height, Precipitation, 2-mspecific humidity , solar radiation	Urban background and rural (1331)	March 15th to April 30th, 2020	Same period in 2015-2019	Generalised Additive Model	In 80% of sites studied NO_2 decreased 5%-55%, and O_3 increased 5%-22%, except in the Iberia Peninsula - which lowered about 7%	In the absence of the lockdown, the NO_2 was expected to raise; the behaviour of O_3 was influenced by the meteorological conditions
[36]	Auckland, New	Study the impact of the lockdown	PM_{10}, $PM_{2.5}$, Black Carbon, O_3, NO_2	24h average	NO_2 (satellite data), T, RH, WS,	Urban (1), suburban roadside (1), and	February to April 2020, being the lockdown	Same period in 2015-2019	t-tests	-The pollutants reduced, except O_3 which increased	The decrease of the vehicular circulation was

	Zealand	on air quality			Rainfall, traffic data	urban background (1)	during March 27th until April 17th			- Black carbon and NO_2 reduced the most	the main cause of air pollution reduction
[90]	China	Evaluate the effects on air quality due to the lockdown and to understand the mechanisms of $PM_{2.5}$ production, in Wuhan, Guangzhou and Beijing	NO_2, SO_2, O_3, $PM_{2.5}$	Daily and weekly average	NO_2, formaldehyde (satellite data)	Monitoring sites from Wuhan (11), Beijing (12) and Guangzhou (12)	5 periods in 2020:<br><br>- P1: January 11th - 17th<br>- P2: January 18th - 24th<br>- P3: January 25th -31st (when lockdown started in some places)<br>- P4: February 1st -7th<br>- P5: February 8th -14th	5 periods in 2019:<br><br>- P1: January 22nd - 28th<br>- P2: January 29th to February 4th<br>- P3: February 5th - 11th<br>- P4 - February 12th-18th; - P5- February 19th -25th<br>And between the 5 periods in 2020	Descriptive Statistics	- NO_2 average concentrations decreased around 28%, 57% and 46%, respectively, in Beijing, Wuhan and Guangzhou, after the lockdown<br><br>- $PM_{2.5}$ levels varied depending on geographical factors<br><br>- For SO_2, little change was notified, whereas O_3 increased significantly during the lockdown, specifically in Wuhan and Beijing	- Formaldehyde registered a slight variation on its concentrations<br><br>- The lockdown was proven to be insufficient to improve air quality
[13]	Lombardy, Italy	Assess the lockdown impact on air quality,	NO_2	Daily average	T, RH, WS, Precipitation	Urban traffic (5), and urban background (1)	2 periods in 2020:<br><br>- Pre-lockdown:	Same period in 2014-2019	Kruskal - Wallis rank sum	NO_2 reduced 4.3%-33.7% based on the scenarios created, which was validated by the decreased	A "modal shift" is proposed in order to reduce air pollution, in a

		using ground-level measurements and scenarios simulations with CAMx					January 1st to March 7th<br><br>- Lockdown: March 8th to April 30th		test, Mann-Whitney-Wilcoxon test	registered with the monitoring sites data	sustainable way
[43]	World wide	Assess the impact of the lockdown on air quality across the 50 most polluted cities	$PM_{2.5}$	Weekly average	Lockdown cities data	Not specified	2 periods in 2020 (varies according to the city in matter):<br><br>- One week in Pre-Lockdown<br><br>- One week during the Lockdown	Comparison between the two periods of measurement	Only Descriptive Statistics	-Bogotá, Colombia, one of the most polluted cities where traffic volume represents 65% of the day demonstrated a significant reduction of $PM_{2.5}$ of about 57%<br><br>- Delhi, the most contaminated city of all, registered a decrease of the $PM_{2.5}$, during the lockdown, of about 40%	- Of all continents, America showed the highest reduction of $PM_{2.5}$ during the lockdown<br><br>- It was highlighted that the data was evaluated attending the population/air quality stations ratio, so a lack of data possibly caused errors and

											misleading on this research
[99]	South Korea	Investigate the impact of the pandemic on air pollution in Seoul and Daegu cities	O_3, NO_2, CO, $PM_{2.5}$, PM_{10}, SO_2	24h average, 8h average (only for O_3)	AQI (calculated), meteorological conditions	Monitoring sites from Seoul (25) and Daegu (15)	January to July 2020	Same period in 2017-2019	Paired t-test, Mann-Whitney U test	- $PM_{2.5}$ diminished in March in both cities around 31%-36%<br><br>- PM registered the main reduction between the lockdown and previous years, in comparison to the gaseous pollutants<br><br>- NO_2, CO and SO_2 registered a partial decrease<br><br>- O_3 levels were not consisting with previous studies	- AQI registered an improvement due to the lockdown<br><br>- A further research of the meteorological data influence is needed to better understand the impact of the lockdown on O_3 behaviour
[41]	USA and China	Assess the changes on air pollution due to the lockdown, and investigate the associatio	NO_2, CO, $PM_{2.5}$, PM_{10}, SO_2	Average concentration	Validated COVID-19 cases and deaths	Not specified	Lockdown periods in 2020:<br><br>- USA: March 19th to April 30th<br><br>- China: January 23rd	Reflected period in 2019	Spearman and Kendall rank correlation tests, Correlation	Overall, the pollutants' concentrations decreased during the lockdown, both in USA and China	NO_2, $PM_{2.5}$ and PM_{10} registered a high association with the COVID-19 disease

		n between COVID-19 virus spread and air pollutants, in some cities from USA and China					to March 29th		coefficient analysis		
[54]	India	Study the changes on air quality during the lockdown in 22 indian cities, and assess the scenario in which similar control on anthropogenic emission sources happens in worst	$PM_{2.5}$, PM_{10}, SO_2, NO_2, CO, O_3, NO, NO_x	Average concentration	AQI [calculated], WS,WD, T, RH	Not specified	March 16th to April 14th 2020	Same period in 2017-2019	Descriptive Statistics	- $PM_{2.5}$ registered the main reduction in all cities<br>- $PM_{2.5}$, PM_{10}, CO, and NO_2 reduced around 43%, 31%, 10%, and 18% during the lockdown when compared to the same period in 2017-2019, whereas O_3 increased by 17%, and no changes were recorded for SO_2	- The increase in O_3 was possibly connected to the decrease of PM and NO_x<br>- AQI diminished significantly, compared to previous years, in all country, around 30%<br>- The excessive risk reduced 4 times due to the air quality improvement

		weather conditions, through WRF-AERMOD								
[48]	India	Assess the impact of the lockdown on air quality in Delhi and Mumbai	NO_2	Daily average	NO_2 (satellite data)	Monitoring sites from Delhi (38) and Mumbai (10)	2 periods in 2020:<br><br>- Pre-lockdown: January 1st to March 24th<br><br>- During and Post-lockdown: March 25th to April 20th	Same period in 2019 and between periods of measurement	Descriptive Statistics	- NO_2 ground-based level reduced by 30 μg/m³- 65 μg/m³ to 12 μg/m³- 25 μg/m³ in Delhi from pre- to post-lockdown period<br><br>- NO_2 diminished as well in Mumbai, resulting in an improvement on air quality<br><br>- Similar findings were obtained with the satellite data	- During March, energy consumption reduced<br><br>- NO_2 emission from shift transportation decreased
[25]	China	Study the impact of the lockdown on air quality	O_3, NO_2, CO, $PM_{2.5}$, PM_{10}, SO_2	Average concentration	N/A	Not specified (1640)	January to April 2020, corresponding to the lockdown period from January 23rd	Same period in 2015-2019	Theil-Sen estimation, Locally Weighted	NO_2, $PM_{2.5}$, PM_{10} and CO decreased 27%, 10.5%, 21.4% and 12.1%, while O_3 had little changes	A further research is needed to assess the impact of the meteorological data on air quality during

						to March 31st, 2020		Scatter plot Smoothing (LOWESS)		the lockdown period
[122]	India	Assess the impact of the lockdown on air quality in Delhi, Kolkata, Mumbai, Chennai, and Hyderabad	$PM_{2.5}$	Average concentration	NO_2 (satellite data), AQI	Monitoring sites from Delhi, Kolkata, Mumbai, Chennai, and Hyderabad (5)	Lockdown: March 22nd – 31st, 2020	Same period in 2019	Descriptive Statistics	Overall, the pollutants' concentrations ($PM_{2.5}$ and NO_2 from satellite data) decreased during the lockdown, especially in Delhi and Kolkata, the two major polluted cities	The lockdown revealed a significant positive impact on air quality on densely populated areas
[61]	India	Determine the impact of the lockdown on air quality in Lucknow and New Delhi	$PM_{2.5}$, SO_2, NO_2, CO	Average concentration	AQI (calculated)	Monitoring sites from Lucknow and Delhi (14)	2 periods in 2020:<br><br>- Pre-Lockdown: February 1st-21st<br><br>- Post-Lockdown: March 25th to April 14th	Comparison between the two periods of measurement	Descriptive Statistics	The pollutants' concentrations decreased during the lockdown, even though the final concentration still surpassed the WHO guidelines	- SO_2 remained almost unchangeable<br><br>- AQI changed from 'moderately polluted'-'poor' to 'good' category

[100]	India	Study the improvement on air quality during the lockdown in Delhi, Kolkata, Mumbai, and Hyderabad	NO, NO_x, NO_2, CO, $PM_{2.5}$, PM_{10}, O_3, SO_2, NH_3, BTX	Average concentration	AQI	Not specified	January to July 2020, with March 25th to May 31st as the lockdown period	Same period in 2019	Descriptive Statistics	- SO_2 and O_3 levels registered an increase in some cities, after the lockdown<br><br>- Overall, during the lockdown, the pollutants' concentrations reduced, increasing with the gradual lifting	In general, AQI reduced during the lockdown by 31%, 73.5%, 31% and 19.5% for New Delhi, Kolkata, Mumbai, and Hyderabad, respectively
[14]	Palermo, Italy	Assess changes on air quality due to the lockdown	CO, NO_2, O_3, PM_{10}	Hourly average, daily average (only for PM_{10})	N/A	Urban Traffic (11)	January 1st to July 31st, 2020	Same period in 2015-2019	Two-tailed paired t-test	CO, NO_2, and PM_{10} reduced around 51%, 50%, and 45% in the lockdown, whereas O_3 increased	O_3 increase due to non-linear chemical effects showed the difficulty in improving air quality during lockdown
[26]	India	Study the influence of the lockdown on air quality in Delhi, Ahmedabad,	$PM_{2.5}$, PM_{10}, NO_2	Daily average	Rainfall, T	City coverage (32-40)	March 20th to April 15th 2020	Same period in: 2013-2019 (Delhi); 2017–2019 (Ahmedabad), 2016–2019 (Mumbai) and 2014–2019 (Pune)	Descriptive Statistics	Overall, NO_2, $PM_{2.5}$, PM_{10} reduced 60%-66%, 25%-50%, and 46%-50%	$PM_{2.5}$ and PM_{10} were mainly reduced in Delhi and Ahmedabad, which suggest wind-blown dust infterference

		Mumbai, and Pune								
[71]	Southern Ontario, Canada	Study the statistical significant changes on air quality due to the pandemic	NO_2, CO, O_3, $PM_{2.5}$	Annual average, daily average	T, radiative forcing	Rural and urban (16)	April, 2020	April 2017-2019	Descriptive Statistics	- NO_2, CO, O_3, and $PM_{2.5}$ decreased around 7%-42%, 11%-20%, 4%-18%, and 0.4%-32%, respectively<br>- In most Southern Cities, O_3 did not show a significant drop	Statistically significant changes occurred when comparing the period measured with the reference data, namely for NO_2 and CO
[109]	Saudi Arabia	Determine the impact of the lockdown on air quality in 9 cities of the country	CO, PM_{10}, SO_2, NO_2, O_3	Average concentration	T, RH, WS	Not specified	January 2019 to February 2020, before the lockdown, and March to May, 2020	Comparison between periods of measurement	ANOVA, Duncan's Multiple Range Test, Students' t-test, Principal Component Analysis	- CO reduced 33.60%<br>- The highest reduction of SO_2 was in Jeddah (44.16%)<br>- PM_{10} registered the largest decrease at Riyadh (91.12%)<br>- NO_2 and O_3 highest reduction were in Makkah (44.35% and 18.98%, respectively)	- Lower concentrations were obtained during the lockdown, with exception of O_3, in Tabuk, Al Qasim, and Haql<br>- Meteorological variations were not significant, with the exception of

										relative humidity in Jeddah
[72]	Wuhan, China	To forecast air quality over a year through PSOSMA-ANFIS model, as well as to evaluate the impact of the lockdown on air quality	$PM_{2.5}$, NO_2, SO_2, CO_2	Not Specified	AQI	Not specified	January 24th to May 8th, 2020, being the lockdown from January 24th until March 8th, 2020	Same period in 2017-2019	MSE, MAE, MAPE, Coefficient of determination	Overall, $PM_{2.5}$, NO_2, CO_2, and SO_2 concentration reduced by 19%, 42%, 22% and 15%, respectively	N/A
[70]	Eastern Province, Saudi Arabia	Evaluate the impact of the lockdown on air quality	NO_2, CO, SO_2, O_3, PM_{10}	Daily average	WS	Monitoring sites from Jubail (1), Qatif (1), Dammam (3), Dammamsouth campus (1) and Al Ahsa (2)	3 periods:<br><br>- Pre-Lockdown: September 15th, 2019, to March 22nd, 2020<br><br>- Lockdown:	Comparison between periods of measurement	Descriptive Statistics	- NO_2 registered the best response to the lockdown: decreased around 12%–86% and 14%–81%, during and after the lockdown, respectively<br><br>- CO reduced drastically between 5.8%-55%, whereas	NO_2, PM_{10}, and SO_2 in some monitoring stations recorded concentrations below the WHO guidelines

							March 23rd to June 20th, 2020<br><br>- Post-lockdown: June 21st to July 18th, 2020			O_3 increased largely, 6.3% to 45%, during the lockdown, and 18% to 263% after it<br><br>- SO_2 decreased (8.7%-30%) during the lockdown, as well as PM_{10} (21% - 70%)	
[27]	China	Study the impact of the lockdown on $PM_{2.5}$	$PM_{2.5}$	Daily average	Air pressure, total column water, wind components, T, total column ozone, RH and planetary boundary layer height, population, and mortality data	Not specified (1388)	Lockdown: February to March, 2020	Same period in 2015-2019	Kolmogorov-Zurbenko filter and multiple linear regression	$PM_{2.5}$ average concentrations decreased around 30%-60%, with the national average concentrations reducing by 18 μg/m^3	- Meteorological factors had negative influence on $PM_{2.5}$ levels in some regions from China, being the anthropogenic emissions the major responsible for the variation of $PM_{2.5}$ levels<br><br>- The avoided premature deaths, in general, corresponded to 9,952, being

										the reduction of the anthropogenic emissions 94 % of the cause of this enhancement.
[123]	Kolkata, India	Assess the changes of air quality during the lockdown and suggest long-term environmentally sustainable measures	NO_2, CO, O_3, PM_{10}, $PM_{2.5}$, SO_2	Diurnal and monthly average	LST variation (LANDSAT 8 OLI and LANDSAT 7 ETM + images), aerosol, NO_2 (satellite data)	Monitoring sites from Fort William, IACS Jadavpur, Rabindra Bharati University, Rabindra Sarobar, and Victoria Memorial	- Lockdown: March 25th to April 15th, 2020	Same period in 2017-2019	Principal Component Analysis, Hierarchical Cluster Analysis	NO_2, $PM_{2.5}$, PM_{10} and CO concentrations were significantly lower during the lockdown period, when compared to the previous years, whereas O_3 revealed an increased tendency	A surface temperature reduction was registered during to the lockdown, when compared to 2017-2019 data
[15]	Vienna, Austria	Study the impact of the lockdown, specifically road transport	NO_2, O_3	Hourly	Total oxidant (O_x), Monthly average daily traffic counts,	Urban traffic, urban background, suburban background, suburban traffic and	February 16th to September 30th, 2020 (lockdown - March 16th to April 13th, 2020)	Same period in 2015-2019	Random forest machine learning algorit	NO_2 reduced around 13.7%-30.4%, while O_3 increased about 3.7%-11.0%	- O_x increased around 1.8%-6.4%<br>- Road traffic reduced especially due to the light-vehicles

		ation changes, on air quality, and the weather conditions influence			mobility data (from Google and Apple), WS, WD, T, P, RH	suburban industrial (17)			hm, Mann-Whitney U-test		- The decrease of pollutants' concentrations was statistically significant
[37]	USA, India, China, and Europe	Assess the impact of the measures implemented on a multi-scale, on air quality	O_3, $PM_{2.5}$, SO_2, CO, PM_{10}, NO_2	Monthly average	NO_2 (satellite data)	Not specified	January to April, 2020	Comparison with 2015-2019	Statistical approach developed by [38]	-The pollutants reduced, except O_3 which increased<br>- In some European cities, besides O_3 other pollutants increased contrarily to other countries – In New Delhi O_3 did not increase	NO_2 from satellite data revealed lower concentrations at a global scale, during the period measured
[73]	Spain	Assess the impact of the lockdown on air quality, in 11 cities	CO, SO_2, PM_{10}, O_3, NO_2	Daily	T, WS, P, Sunlight hours and atmospheric pressure	Traffic stations (11)	March 2nd to April 12th, 2020, with the "minor lockdown" on March 15th-19th, and the "major lockdown" on March	March 4th to April 14th, 2019	Linear regression	In some cities, NO_2, SO_2, CO and PM_{10} registered a reduction of the concentration, whereas O_3 increased	The improvements on air quality, due to the lockdown, were not significant

							19th to April 12th				
[74]	Istanbul, Turkey	Assess the impact of the partial-lockdown on air quality	PM_{10}, SO_2, CO, NO_2, NO, NO_x, O_3	Average concentration	N/A	City coverage (19)	2 periods in 2020:<br><br>- Pre-Lockdown: March 1st to April 9th<br><br>-Partial-Lockdown: April 10th to May 22nd	Same period in 2019 and comparison between the periods of measurement	Descriptive Statistics	- Overall, the pollutants' concentrations decreased during the partial-lockdown relatively to the previous year and to the pre-lockdown period<br><br>- O_3 concentrations in some areas registered an increase, with the maximum increase of 61.67%	Further research is needed, accounting the meteorological and industrial influence, which significantly impact the air quality
[124]	India	Assess the impact of the lockdown on air quality	$PM_{2.5}$	Daily	$PM_{2.5}$, NO_2, SO_2, O_3 (satellite data), CH_4, formaldehyde, CO, T, station pressure, RH	Monitoring sites from Delhi, Kolkata, Mumbai, Chennai, and Hyderabad (5)	2 periods in 2020:<br><br>- Pre-Lockdown: March 10th-21st<br><br>- Lockdown: March 22nd-31st	Same period in 2019	Descriptive Statistics	$PM_{2.5}$ reduced in all cities, with exception for Chennai, where no significant changes occurred	The gases studied registered a decrease in their concentrations, with particular focus on NO_2 which registered lower levels than those from 2019

[91]	Tunisia	Study how the the lockdown affected the air quality in Tunis, Sousse, Sfax, and Tataouine	CO, NO_2, O_3, PM_{10}, $PM_{2.5}$, SO_2	Monthly average	AQI	Not specified	January to April 2020, in which first measures were imposed on March 12th	Comparison between periods of measurement	Descriptive Statistics	- The $PM_{2.5}$, NO_2, SO_2, and CO concentration decreased significantly, whereas O_3 increased<br><br>- It is highlighted that in Tataouine, PM_{10} increased, whereas in the majority no significant changes were observed<br><br>- Overall, NO_2, $PM_{2.5}$, SO_2 and CO decreased between 22%-51%, 7%-23%, 18%-52% and 4%-19%, respectively, during March compared to January	Based on AQI, air quality improved in the four cities studied
[28]	China	Evaluate the impact of the lockdown on air quality in Wuhan, Hubei,	$PM_{2.5}$,PM_{10}, SO_2, NO_2, O_3, CO	Daily average	N/A	Not specified (365)	January 21st to March 23rd 2020	Same period in 2015-2019	Descriptive Statistics	- NO_2 reduced 53%, 50% and 30%, in Wuhan, Hubei and China, as well as $PM_{2.5}$ by 35%, 29% and 19%, when compared to 2019	N/A

		and China (excluding Hubei)								- PM_{10} had similar reduction to $PM_{2.5}$<br>- SO_2 and CO reduced but not as much as the before-mentioned pollutants<br><br>- O_3 increased up to 58%, in Wuhan	
[125]	Indo-Gangetic Plain (IGP), India	Assess the impact of the lockdown on air quality on the ten most polluted cities from IGP	$PM_{2.5}$	Weekly and monthly average	T, Rainfall, WS, RH	Not Specified	3 periods in 2020:<br><br>- Pre-lockdown: January 1st to March 24th<br><br>- Lockdown: March 25th to May 31st<br><br>-Post-lockdown (1º phase): June 1st-30th	March to June, 2017-2019 and between periods of measurement	Pearson's correlation	- $PM_{2.5}$ concentration on the 1st week of January was about 284 μg/m^3, decreasing more than 65% within the first 15 days of the lockdown<br><br>- During and post-lockdown periods, the $PM_{2.5}$ concentration reduced around 34% in comparison with the same period in 2017-2019.	- Air quality improved during the lockdown<br><br>- Long-term lockdown is not an option to improve air quality, because of other political questions
[126]	Indo-Gangetic Plain	Study the impact of the lockdown	$PM_{2.5}$	Monthly average	T, WS, Rainfall, RH	Not specified	January 1st to May 17th 2020	Between pre- and during the lockdown and with the	Coefficient of Variation,	- $PM_{2.5}$ decreased, in comparison to 2019, about 26.38% - 36.75%	$PM_{2.5}$ concentration decreased significantly

	(IGP), India	on air quality in 12 IGP cities						same period in 2018-2019	ANOVA	- Overall, the $PM_{2.5}$ concentrations reduced significantly	just after 1 week of the lockdown, therefore a 3-7 day lockdown could be a possible measure to implement, to improve air quality
[127]	India	Assess the impact of the lockdown on air quality in Delhi and Kolkata	NO_2, NO, PM_{10}, $PM_{2.5}$, O_3	24h average	WS, T, WD	Background stations from Delhi (5) and Kolkata (2)	2 periods in 2020:<br><br>- Pre-lockdown: March 1st-21st<br><br>- Lockdown: March 22nd to May 3rd	Same period in 2019	ANOVA	- During the lockdown, PM_{10} reduced by 59% and 49% in Delhi and Kolkata, respectively. $PM_{2.5}$ diminished 43% and 50%, respectively in comparison with 2019 data<br><br>- The O_3 concentration increased in Kolkata, from 31.4 μg/ m^3 to 46.9 μg/ m^3 relatively to 2019, whereas in Delhi it decreased significantly from	- During the lockdown period, the air quality improved<br><br>- PM were highly affected by the lockdown, while O_3 behaviour had to do with spatial variations

									44.7 µg/ m³ to 38.5 µg/m³	
[16]	Southern Italy	Study the impact of the lockdown on air quality, namely size and concentration of submicron particles	Submicron particles	Daily average	T, RH, Rainfall, WS, WD, size particles data	Urban background (1), - suburban (1)	3 periods in 2020: - Pre-Lockdown: January 1st to March 9th - Lockdown: March 10th to May 17th - Post-Lockdown: May 18th to July 31st	Same period in 2015-2019	Mann-Whitney U-test	Submicron particles reduced about 4% to 23%.	Air quality improved in only one of the studied sites
[110]	Valencia, Spain	Determine the impact of the lockdown on air quality and pollutants' emission	PM_{10}, $PM_{2.5}$, NO_x, NO_2, NO, O_3	Daily average	WS, Rainfall, RH, T, P, solar irradiance	Urban traffic (4), suburban traffic (1), urban background (1), suburban (background)	6 periods in 2020: - Pre-Lockdown: January 1st to March 14th - Lockdown: March 15th to May 17th	Same periods in 2019	Paired t-test	- PM_{10} and $PM_{2.5}$ registered decreases from 41% to 60% in three urban stations - NO, NO_x, NO_2 registered reductions from 35.3% to 67.7% in all sites - O_3 also registered a decrease in its concentration	N/A

							- Phase 1: May 18th-31st<br><br>- Phase 2: June 1st-14th<br><br>- Phase 3: June 15th-20th<br><br>- Post-Lockdown: June 21st to September 30th				
[75]	Novi Sad, Republic of Serbia	Assess the impact of the lockdown measures on air quality and their health benefits in urban areas	SO_2, NO_2, O_3, PM_{10}, $PM_{2.5}$	Average daily concentration, average daily max 8h	T, RH, P, wind velocity, total mortality data	Urban traffic, suburban background, suburban traffic, suburban industrial, urban background (all monitoring sites)	2 periods in 2020:<br><br>- Pre-Lockdown: January 1st to March 14th<br><br>- Lockdown: March 15th to May 6th	Same period in 2018-2019	t-test, ANOVA,	- Overall, the pollutants' concentrations decreased, while O_3 concentration increased<br><br>- $PM_{2.5}$ registered the most significant reduction	The significant reduction of $PM_{2.5}$ led to a high number of avoided deaths

[60]	Sydney, Australia	Study the effects of the lockdown on air quality, with ground-based data and with WRF-CMAQ model, as well as to study the sensitivities of air pollutants to meteorological and emissions changes	CO, NO_2, $PM_{2.5}$, O_3	Average concentration, hourly	O_3, NO_2, SO_2, aerosols (satellite data), Traffic data	Not specified	April to June, 2020	Same period in 2016-2019	Generalised Linear Model, Welch's t-test	NO_2, $PM_{2.5}$ and CO registered a decrease in their concentrations, while O_3 increased.	The results obtained with the WRF-CMAQ model were consisting with the determined with the ground-based data.
[104]	India	Study the impact of the lockdown on air quality with	NO_2, CO, SO_2, O_3, NH_3, $PM_{2.5}$, PM_{10}, NO	Daily average	AOD, NO_2 (satellite data), T, RH, WS, Precipitation	Monitoring sites from urban, suburban, rural, residential,	3 periods in 2020:<br><br>- Pre-lockdown: March 1st - 24th	Same period in 2017-2019 and between periods of measurement	Descriptive Statistics	- Overall, NO, NO_2, $PM_{2.5}$, PM_{10}, SO_2 and CO concentrations diminished around 27%-64%, 27%-47%, 24%-29%, 7%-26 %,	- The increase of O_3 resulted from NO reduction and higher photolysis rates, this

	particular focus on Delhi-National Capital Region, and to simulate the changes on air pollutants' levels in two different scenarios, with the WRF-CHIMERE		and traffic areas (63)	- Lockdown: March 25th to May 17th<br><br>- Post-lockdown: May 18th to June 4th		10%-11% and 9%-28%, respectively<br><br>- As for the O_3, in Delhi-NCR, it increased 4%-7%	lasted due to air pollution and aerosol reduction (by 30%-50% in the Northern India)<br><br>- WRF-CHIMERE model simulations revealed an air pollution decrease, over all India, namely in $PM_{2.5}$, NO_2 and SO_2, by 20%-48%, 55%-92% and 56%-85%, respectively. These reductions were largely higher than those obtained from the monitoring sites data

										-The pollutants' levels increased during the post-lockdown period
[76]	Poland	Study the effect of the lockdown on air quality	$PM_{2.5}$, PM_{10}, NO_2, SO_2	Daily average	AOD, NO_2 (satellite data)	Monitoring sites located on urban, suburban, rural, and industrial areas (28)	March to May 2020, with lockdown on March 15th until April 25th	Same period in 2018-2019	Descriptive Statistics	- Overall, the highest decrease registered was for $PM_{2.5}$ and PM_{10}, during April and May, compared to 2019<br><br>- $PM_{2.5}$ reduced between 11.1%-26.4% and 8.7%-21.1%, in April and May, respectively<br><br>- PM_{10} diminished around 8.6%-33.9% and 8.5%-31.5%, in April and May, accordingly	AOD decreased by 23% and 18%, in comparison to the 2018-2019 period, respectively in April and May, whereas NO_2 reduced by 10% and 19%, respectively
[111]	South China	Assess the impact of the lockdown on air quality in	CO, NO, NO_x, NO_2, O_3, PM_{10}, $PM_{2.5}$, SO_2	Hourly, daily	WS,WD, precipitation, RH, T, P, visibility, AQI	Not specified (50)	3 periods, subdivided, in 2020:<br><br>- Pre-lockdown	Same period in 2016-2019	Multiple Linear Regression Model	Criteria pollutants registered a decrease in the Guangxi region, as well as O_3, which decreased due to 34.4% synergistic	- The decrease of NO_x proved to stimulate the reduction of O_3

	14 cities from South China (Guangxi), and to quantify the influence of meteorological conditions and anthropogenic contributions, and to determine the effects of local sources and long-range transport by a continuous emission monitoring system		- 1st phase lockdown: Spring Festival, Level I lockdown, and Level I restoration (February 10th)<br><br>- 3rd phase of the lockdown: February 25th to April 30th (recovery), and May - June (operation)		effects of the lockdown and 65.6% due to weather effects, according to the MRL results	- Overall, an improvement on air quality was registered due to the lockdown

		and HYSPLIT model								
[17]	Portugal	Assess the impact of the lockdown on air quality	NO_2, PM_{10}	Hourly, daily average	Mobility data	Rural (9), urban background (14) and urban traffic (11)	2 periods, in 2020:<br>- Lockdown: January 1st to March 15th<br>- Lifting: March 16th to May 31st	Comparison between the two periods of measurement and the same period in 2015-2019	Descriptive Statistics	- NO_2 and PM_{10} diminished around 41% and 18%, with NO_2 reduction above 60% on urban areas<br>- Light increase on NO_2 and PM_{10} concentration was noticed in the last 2 weeks of May	- Transportation reduced by 50%-70%, during lockdown<br>- The increment method used for the approach of mobility activity revealed little consistency for the estimation made
[77]	India	Assess the impact of the lockdown phases on air quality in Delhi, Mumbai,	PM_{10}, NO_2	Daily average	AQI (calculated)	Background, Urban	2 periods in 2020:<br>- Pre-Lockdown: February 14th to March 24th	Same period in 2019	Paired t-test	- Overall, PM_{10} and NO_2 reduced, revealing higher decreases in the urban sites than in background	More monitoring sites in Mumbai and Kolkata are required, in order to obtain a more

		and Kolkata					- Lockdown: March 25th to May 3rd			- NO_2 registered higher reductions than PM_{10}	representativeness of changes on air quality
[56]	China	Assess and quantify the impact of the lockdown on air quality in Wuhan, Beijing, Shanghai, and Guangzhou, as well as to identify the probable sources of $PM_{2.5}$ emissions using the HYSPLIT and CWT models	$PM_{2.5}$, SO_2, NO_2	Monthly average	P, WS, T, RH, Boundary Layer Height, PVPT, PVT	Monitoring sites from each megacity (40-48)	6 periods in 2020:<br><br>- Pre-lockdown: January 1st-22nd<br>- P1: January 24th to February 8th<br>- P2: February 9th-29th<br>- P3: March<br>- P4:April<br>- P5: May	Same period in 2016-2019 and between pre- and during the lockdown	Wilcoxon test, stepwise regression with backward elimination, Spearman correlation	- In Wuhan SO_2 registered an increase, and in Beijing $PM_{2.5}$ concentration also increased<br><br>- In general, all pollutants showed a reduction, broadly satisfying the Environmental Air Quality Standard of China guidelines<br><br>- NO_2 demonstrated to be the most sensitive pollutant to the effect of the lockdown measures	- Traffic volume showed a strong correlation with NO_2, and the meteorological data with $PM_{2.5}$<br><br>- National-level measures need to be implemented in order to reduce and control NO_2 emission sources, with particular focus on traffic sources.

[29]	National Capital Regional, India	Assess the impact of the lockdown on air quality	PM_{10}, $PM_{2.5}$ NO_x, NO, NO_2, NH_3, SO_2, CO, Benzene, O_3	24h average	RH, T, WS, solar radiation, AQI (calculated)	Monitoring sites from Delhi (20), Gurugram (4), Faridabad (4), Ghaziabad (4), and Noida (4)	March 1st to May 1st 2020, with the lockdown on March 25th to April 1st	Comparison between 8 days before (March 17th - 24th) and 8 days during the lockdown; between 24 days before and after the lockdown; and with average concentrations from January 1st to 30th November, since 2015 until 2019	Pearson's correlation, ANOVA	PM_{10}, $PM_{2.5}$, NO_x, NO, NO_2, SO_2, CO, NH_3 and Benzene reduced around 61.6%, 60.0%, 58.6%, 62.3%, 46.8%, 33%, 44.8%, 26.6% and 53%	- O_3 increased in later days of the lockdown due to the increase of solar radiation and ambient temperature<br>- A more significant reduction occurred in the 8 days before the lockdown, than in the 24 days before and after the lockdown
[62]	USA	Determine the impact of the lockdown on air quality and study the spatio-	$PM_{2.5}$	Daily average	T, WS, P, snow	Not specified	January 1st to June 29th, 2020	Comparison between periods of measurement	Linear mixed effects model, Functional concurrent regress	$PM_{2.5}$ concentrations reduced during the lockdown relatively to the pre-lockdown across the 50 states evaluated, being as low as the measures implemented were more restrictive	N/A

		temporal heterogeneity						ion model		
[89]	Baghdad, Iraq	Assess the impact of the lockdown on air quality	NO_2, PM_{10}, O_3, $PM_{2.5}$	Daily average	NO_2 (satellite data), AQI (both sites data and calculated)	Not specified	5 periods in 2020:<br><br>- Pre-lockdown: January 16th to February 29th<br><br>- P1 (1st partial and total lockdown): March 1st to April 21st<br>- P2 (2nd partial lockdown): April 22nd to May 23rd<br>- P3 (2nd total lockdown): May 24th to June 13th<br>- P4 (3rd partial lockdown):	Comparison between the pre- and lockdown periods (P1, P2, P3, P4)	Descriptive Statistics	- NO_2 reduced 6%, 7%, 8% and 20%, during P1, P2, P3, and P4, respectively<br><br>- $PM_{2.5}$ and PM_{10} demonstrated a decrease by 8% and 15%, respectively, during P1<br><br>- O_3 increased by 13%, 75%, 225% and 525%, during P1 , P2, P3 and P4, respectively, potentially caused by the NO_2 and PM_{10} reduction	- NO_2 levels (from satellite data) reduced by 35%-40%, during the lockdown<br>- AQI enhanced around 13%, during P1, even though temporarily<br>- Even though $PM_{2.5}$ and PM_{10} concentrations decreased, they remained above WHO guidelines

							June 14th to July 24th				
[49]	Mexico City Metropolitan Area, Mexico	Determine the influence of the lockdown phases on air quality and their public health benefits	CO, NO_2, O_3, SO_2, PM_{10}, $PM_{2.5}$	Daily average	NO_2, CO (satellite data), AQHI ASI, Mobility data	Urban, industrial, commercial, residential, and urban background	Lockdown in 2020:<br>- Phase 2: March 17th to April 20th<br>- Phase 3: April 21st to May 31st	Same period in 2016-2019	Fourier series	Overall air quality improved with the pollutants' concentrations decrease, with exception of O_3 whose concentration increased	The improvements on air quality due to the imposed lockdown measures lead to 588 avoided-deaths due to air pollution
[59]	India	Study the impact of the lockdown on aerosols, radiation and trace gases and their correlated chemistry	Black Carbon, $PM_{2.5}$, PM_{10}, NO, NO_2, CO, O_3, SO_2,	Daily average	AOD, NO_2 (satellite data), T, RH, WS, Atmospheric Boundary Layer height, PM_1, CO_2, CH_4	tropical rural site (1)	6 periods in 2020:<br><br>- Pre-Lockdown: February 15th - March 21st;<br><br>- Total Lockdown: Phase I : March 25th - April 14th Phase II: April 15th - May 3rd Phase III :	Same period in 2019, and comparison between the periods of measurement for PM	Descriptive Statistics	Black carbon, PM_1, $PM_{2.5}$, PM_{10}, NO, NO_2, CO, O_3, SO_2 reduced 34.2%, 40.9%, 46.7%, 50.4%, 55.4%, 58.8%, 10.1%, 7.1%, and 63.7%, respectively, during the lockdown in comparison with 2019	- During the third phase of the lockdown, it was registered the maximum phase-wise changes<br><br>- CO_2 and CH_4 increased about 0.8% and 1.1%, respectively, during the lockdown when

							April 4th - May 17th Phase IV: May 18th - May 31st				compared to 2019<br><br>- NO_2 from satellite data reduced about 30.2%, as well as AOD about 22.9%<br><br>- AQI remained in the same category, revealing that the air quality in Gandaki area did not change
[94]	India	Study the impact of the lockdown on a social, economic, and environmental - namely	$PM_{2.5}$, PM_{10}, NO_2, SO_2, NH_3, CO, O_3	Daily average	AQI, Market data, Confirmed COVID-19 cases	Suburban (1), Industrial (2), and Urban (9)	2 periods in 2020:<br><br>- Pre-Lockdown: 40 days before the lockdown<br><br>- Lockdown (Phase I	Comparison between periods of measurement	Student's t-test, Pearson's correlation	- $PM_{2.5}$, PM_{10}, NO_2, and O_3 reduced during the lockdown to below the standard limit imposed by Central Pollution Control Board and the Ministry of Environment and Forest	AQI improved

		air quality - domain					and Phase II): 40 days			- NH_3, SO_2, and CO, even during the pre-lockdown period, fulfilled the standard limit imposed	
[34]	Mexico	Study the impact of the the lockdown on air quality	SO_2, NO_2, CO, PM_{10}, $PM_{2.5}$, O_3	Average concentration	Average traffic count, T, RH, WS, Precipitation	Not specified	2 periods in 2020: - Pre-lockdown: January 1st to March 31st - Lockdown: 1st phase: April 1st-30th 2nd phase: May 1st-31st	Same period in 2015-2019 and comparison between the 1st lockdown phase with pre-lockdown, and between the two lockdown phases in 2020	Correlation tests	- Compared to the pre-lockdown period, SO_2, NO_2 and PM_{10} reduced by 55%, 29% and 11%, whereas O_3, CO and $PM_{2.5}$ increased around 63%, 1.1% and 19%, respectively - In comparison to the 2015-2019, NO_2, SO_2, CO, PM_{10} and $PM_{2.5}$ reduced by 19%-36%, and O_3 decreased around 14%	- Air quality improved during the second phase of the lockdown - The pollutants behaviour proved to be related with traffic emissions reduction, and not with the weather conditions

										- $PM_{2.5}$, O_3 and CO showed significant correlation with COVID-19 cases and deaths<br><br>- Pollutants' concentrations would increase with the lifting
[35]	California, USA	Assess the changes on air quality due to the lockdown	NO_2, O_3, $PM_{2.5}$, PM_{10}, CO	Daily average	NO_2 (satellite data), main power plants, highways, and<br><br>wildfire's location	Not Specified	3 periods in 2020:<br><br>- Pre-lockdown: January 26th to March 18th<br><br>- Lockdown: March 19th to May 8th<br><br>- Post - lockdown:	Same period in 2015-2019 (annual means) and between the periods of measurement	Pollutants' concentrations Normalization	- CO reduced more than NO_2 and $PM_{2.5}$ during lockdown<br>- NO_2 increased in residential and transportation hub areas.	- The spatiotemporal patterns were influenced by lockdown<br>- During the post-lockdown period, the air pollutants increased compared to previous years

						May 9th to June 14th				
[18]	Po Valley, Italy	Study the effects of the lockdown, namely the anthropogenic emissions' reduction, on air quality	NO_2, Benzene, NH_3	Monthly average, daily average	N/A	Monitoring sites selected for NO_2 (218), Benzene (62), and NH_3 (14) from Emilia-Romagna, Lombardia, Piemonte, and Veneto	January to June 2020	Same period in 2014-2019	Kolmogorov-Smirnov test	- NO_2 and benzene (traffic-related) decreased about 35%-40%<br>- NH_3 (agriculture-related), did not significantly changed	Lockdown had higher impact in the traffic areas, and on the traffic-related pollutants

[19]	Graz, Austria	Assess the influence of the lockdown on air quality	O_3, PM_{10}, NO_2	Average concentrations	Traffic data, total oxidant (O_x), T, RH, P, WS, WD, precipitation	Traffic, industrial, urban background (5)	January to May 2020	Same period in 2014-2019	Principal Component Analysis, Random Forest Regression	PM_{10} and NO_2 decreased during lockdown, whereas O_3 increased	PM_{10} and NO_2 predicted levels were higher than those verified during the lockdown, contrarily to the O_3 (predicted levels above the real levels)
[112]	Delhi, India	Assess the impact of the lockdown measures and post-lockdown on air quality and their health benefits	PM_{10}, $PM_{2.5}$, NO_2, O_3	Daily average	Meteorological data	Industrial, Residential, Commercial (11)	2 periods in 2020:<br>- Lockdown:<br>Phase 1: March 25th to April 14th<br>Phase 2: April 15th to May 3rd<br>Phase 3: May 4th-17th<br>Phase 4: May 18th-31st<br><br>- Post-Lockdown:	Same period in 2018-2019	Paired t-test	- PM_{10}, $PM_{2.5}$, and O_3 reduced about 61%, 48%, and 42%, respectively, during the first phase of the lockdown, while NO_2 registered a higher decrease during the second phase of the lockdown (around 49%)<br><br>- The pollutants' concentrations increased in the post-lockdown period, nonetheless PM_{10}, $PM_{2.5}$, NO_2, and O_3 registered a	The reduction of O_3 and $PM_{2.5}$ led to 903 avoided premature deaths due to air pollution, which correspond 60% less than the previous years

							Phase 1: June 1st-30th			reduction of 40%, 30%, 24%, and 14%, respectively, relatively to 2018-2019<br><br>- Higher reductions were found in traffic and industrial areas, except for O_3	
[20]	Italy	Assess the impact of the restrictive measures applied on air quality	PM_{10}, $PM_{2.5}$, NO_2	Weekly average	N/A	Not specified	February 24th to May 4th, 2020	Same period in 2014-2019	Panel regression	- PM_{10} and NO_2 decreased about 5.125 μg/m^3 and 5.375 μg/m^3<br><br>- $PM_{2.5}$ did not statistically significant changed	The lockdown measures cannot assure long-term improvement on air quality
[115]	Reggio Emilia, Italy	Assess the impact of the lockdown in both road vehicular traffic and air quality	NO_2, CO, O_3, PM_{10}, $PM_{2.5}$	Annual, daily average	T, WS, WD, P, Vehicle traffic data	Urban background (1) and urban traffic (1)	February 1st to May 29th, 2020, with the lockdown period on March 10th to May 18th	Same period in 2019	Descriptive Statistics	NO_2 registered the highest decrease (32% at the urban traffic area, and 41% at the urban background station), followed by CO with 22% overall<br><br>- PM_{10} and $PM_{2.5}$ concentrations increased around 23% (urban	- Vehicles circulation reduced up to 82% during the lockdown, in comparison with the reflected period in 2019<br><br>- The improvement in air quality

									background), 27% (urban traffic), and 31% (urban background), respectively<br><br>- O_3 in the urban background station increased 13%	was not entirely by the decrease of the vehicles circulation, since meteorological conditions played an important role in the air quality and dispersion of the pollutants
[119]	Pakistan	Study the impact of the different periods of the lockdown and lifting, on air quality in Lahore, Karachi, Peshawar, and Islamabad	$PM_{2.5}$	24h average	NO_2 (satellite data), COVID-19 cases, AQI (calculated)	Not specified (4)	4 periods in 2020:<br><br>- Pre-lockdown: January 1st to March 21st/April 2nd<br><br>- Lockdown: March 22nd/April 2nd to May 9th/May 30th	Between periods of measurement	Pearson's correlation	The maximum average concentration of $PM_{2.5}$ registered was around 108 μg/m^3, in Lahore, four times higher than the WHO guidelines	- AQI remained in the "very unhealthy" category in all the cities during the four periods of measurement<br><br>- Pearson's correlation revealed a poor association between the air pollutants

							- Relaxation period: May 10th/June $1^{st}$to June 16th/June 17th<br><br>- Smart-lockdown: June 17th/June 18th to June 30th (period of measurement depending on the city in matter)				(city-level) and COVID-19 cases (province-level)<br><br>- No significant improvement was registered from the NO_2 satellite data images
[103]	Chandigarh, India	Investigate the impact of the lockdown on air quality	$PM_{2.5}$, PM_{10}, NO_2, NO, NO_x, SO_2, O_3, NH_3, CO, Benzene, Toluene, o-Xylene, m-Xylene, p-Xylene,	Daily average	T, RH, WS, WD, Solar insolation, Rainfall	Not specified	2 periods in 2020:<br><br>- 21 days before the lockdown<br><br>- March 25th – May 17th, divided	Comparison between pre-lockdown period and during lockdown	Pearson's correlation, Principal Component Analysis	All pollutants showed a significant reduction with exception for SO_2, O_3 and m,p-xylene	- The reduction found was associated with the interruption of the traffic and industrial activities in the two phases of the lockdown, whereas all

			Ethylbenzene				into 3 sub-periods				pollutants' concentrations increased in the third phase, when the activities mentioned began<br><br>-Vehicular emissions contributed primarily for the air pollution, along with the local residential emissions
[63]	Makkah, Saudi Arabia	Study the changes on air quality due to the lockdown, and the spatiotemporal variations of the air	SO_2, NO_2, CO, O_3, PM_{10}	Daily average, hourly average	WS, WD, RH, T, Rainfall	City coverage (6)	2 periods in 2020:<br><br>- Pre-Lockdown: January 1st to March 10th<br><br>- Lockdown: March 11th	Comparison between periods of measurement	Descriptive Statistics	SO_2, NO_2, CO, O_3, and PM_{10} reduced from 7.27 to 5.31 μg/m^3, 18.10 to 13.48 μg/m^3, 0.93 to 0.67 mg/m^3, 26.76 to 24.77 μg/m^3, and 143.0 to 111 μg/m^3, respectively, during the lockdown relatively to the pre-lockdown period	The traffic activities counted 25%-30% of the air pollution in Makkah city, which were the main cause of the change on air pollutants' concentration

		quality indicators					to August 31st				
[51]	India	Study the impact of the lockdown on air quality, in the 14 cities most sensitive to the COVID-19	NO_2, O_3, SO_2, PM_{10}, $PM_{2.5}$	Monthly average	AQI, NO_2 (satellite data)	Not specified (200)	2 periods in 2020:<br><br>- Pre-lockdown: February 25th to March 24th<br><br>- During and Post-lockdown: March 25th to April 24th	Between periods of measurement	Linear regression, Correlations analysis	- Overall, NO_2, PM_{10} and $PM_{2.5}$ concentrations decreased around 48.68%, 34.84% and 33.89%, respectively<br><br>- NO_2 levels from satellite data also decreased	Positive correlations between COVID-19 mortality and AQI and PM_{10} were found
[113]	Hanoi, Vietnam	Assess the impact of the partial-lockdown on air quality	$PM_{2.5}$, SO_2, NO_2, O_3	Daily average	$PM_{2.5}$ (from sampling sites), Precipitation, T, Boundary Layer Height, dew point temperature, surface solar	Not specified	2 periods in 2020:<br><br>- Before Partial-Lockdown: March 10th-31st<br><br>- Partial-Lockdown: April 1st-22nd	Same period in 2014, 2016, and 2017	Principal Component Analysis	- NO_2, $PM_{2.5}$, O_3, and SO_2 decreased by 75.8%, 55.9%, 21.4%, and 60.7%, respectively, relatively to the reference years<br><br>- O_3 increased by 40.7% during the partial-lockdown, compared to the period before it	$PM_{2.5}$ concentrations from the sampling sites decreased about 41.8%, being consistent with the reduction obtained with the ground-based levels

					irradiation, RH (calculated)					
[21]	Turkey	Assess the impact of the lockdown on air quality in 81 cities from Turkey	PM_{10}, SO_2	Daily average	Mobility data, Car-purchasing data	Not specified (minimum of 81 sites)	January to November, 2020	Same period in 2015-2019	Welch's t-test, F-test, Pearson's correlation	- PM_{10} reduced 53.90 μg/m³- 43.75 μg/m³ during the lockdown<br>- SO_2 increased slighlty in the lockdown and significantly in the post-lockdown	N/A
[96]	India	Study the impact of the lockdown on air quality, land surface temperature and anthropogenic heat flux	PM_{10}, $PM_{2.5}$, SO_2, CO, NO_2, NH_3, O_3	Daily	LST, AHF, PM_{10} (satellite data), AQI (calculated)	Not specified	April 24th to May 25th, with the beginning of the lockdown on March 28th	Same period in 2018-2019	Pearson's correlation	The pollutants' concentrations reduced during the lockdown, with PM_{10} decreasing from 102 μg/m³ to 18 μg/m³	- LST decreased by 4.02 ºC, and AHF reduced from 116 W/m² to 40 W/m², during the lockdown<br><br>- AQI registered an improvement, increasing from poor-very poor category to moderate-satisfactory

[106]	Mexico	Study the impact of the lockdown on air quality	NO_2, CO, NO_x, O_3	Hourly average	CO, formaldehyde, NO_2 (satellite data), WS, ultraviolet rays, RH, population data	Monitoring sites from northeast, northwest, centre, southeast and southwest urban areas (5) and all the measurement sites of the mentioned regions	March 1st to - May 31st	Same period in 2018 -2019	t-test (two tail)	NO_x reduced around 30% compared to the previous years, while O_3 concentrations increased	- CO, NO_2 and formaldehyde from satellite data decreased by 7%, 19% and 7%, respectively, in comparison to the average data from the previous years
[79]	India	Study the impact of the lockdown and lifting on air quality, in Chennai, Hyderabad, Kolkata, Mumbai, and New Delhi	$PM_{2.5}$	Daily average	Rainfall, RH, T	Not specified	3 periods in 2020:<br><br>- Pre-lockdown: March 1st-24th<br><br>- Lockdown: March 25th to May 31st<br><br>- Post-lockdown:	Same period in 2016-2019	Descriptive Statistics	$PM_{2.5}$ reduced during the lockdown around 62%, 49%, 34%, 26% and 10%, in Kolkata, Mumbai, Chennai, New Delhi and Hyderabad, respectively	- In the post-lockdown period, $PM_{2.5}$ concentrations met the national standards<br><br>- Weather conditions influenced the reduction of the $PM_{2.5}$ concentrations

							June 1st to August 31st				
[80]	Delhi Metropolitan Region, India	Assess the effects on air quality due to the lockdown, in terms of pollutants' concentrations and spatial patterns	PM_{10}, $PM_{2.5}$	Monthly average, hourly average	N/A	Not specified (34 stations for PM_{10} and 31 for $PM_{2.5}$)	April 2020	Comparison with April 2019	Simple Kriging	PM_{10} and $PM_{2.5}$ reduced about 20%-70% and 15%-90%, respectively	Spatially, the pollutants' concentrations decreased the most in the downtown and industrial areas of the Delhi Metropolitan Region
[52]	India	Evaluate the improvement on air quality due to the lockdown, in Punjab state and Chandigarh, and to associate COVID-19 confirmed cases with	NO_2, CO, $PM_{2.5}$, PM_{10}, O_3, SO_2	Average concentration	AQI, T, WS, RH, Rainfall, COVID-19 daily cases	Not specified	3 major periods in 2020:<br><br>- Pre-lockdown: March 1st-24th<br><br>- Lockdown: 1st phase: March 25th to April 14th	Comparison between periods of measurement	Spearman's Rank Correlation	- Overall, in relation to the pre-lockdown period: $PM_{2.5}$ reduced by 52%, in the 1st phase, and up to 40%, in the 2nd, and PM_{10} reduced by 53% and 28%, in each phase, respectively.<br><br>- In the final phases of the lockdown, and post-lockdown, PM levels increased	- A temporary lockdown could be an effective way to implement in order to improve the air quality<br><br>- The meteorological conditions did not prove to be highly associated with the

		the meteorological conditions and atmospheric pollutants' concentrations					2nd phase: April 15th to May 3rd<br>3rd phase: May 4th-17th<br>4th phase: May 18th-31st<br><br>- Post-lockdown:<br>1st phase: June 1st-30th<br>2nd phase: July 1st-10th			- During the 4 phases of the lockdown, NO_2 concentrations remained below 20 μg/m^3 (being 80 μg/m^3 the limit value), increasing in the post-lockdown period. CO also reduced significantly, whereas O_3 and SO_2 registered an increase	COVID-19 disease, however the study demonstrated that PM could possibly play as a carrier to spread the coronavirus
[67]	Maharashtra, India	Study the impact of the lockdown and lifting on air quality	NO_x, NO, NO_2, CO, O_3, PM_{10}, $PM_{2.5}$, SO_2, Benzene, Toluene	Daily average	AQI (calculated), COVID-19 cases data, T, Rainfall, WS	City coverage (22)	3 periods, with different phases, in 2020:<br><br>- Pre-Lockdown : 1) January 1st-31st ; 2) February 1st-29th; 3) March 1st-24th	Between post- and during the lockdown periods with the pre-lockdown	Kendall rank correlation, Exploratory Data Analysis and Saphiro's Wilk's test, Kruskal-Wallis	- Overall, the pollutants' concentration reduced, specifically $PM_{2.5}$ and PM_{10} decreased by 51% and 46%, respectively during the lockdown. As for O_3 and SO_2, these pollutants' concentration registered little changes during the lockdown	- AQI stayed in the "Satisfactory" category during the lockdown, diminishing during the post-lockdown<br><br>- Higher concentrations on $PM_{2.5}$ and PM_{10} could lead to higher chance of

							- Lockdown: 1) March 25th to April 14th; 2) April 15th to May 3rd ; 3) May 4th-17th 4) May 18th-31st<br><br>- Post-Lockdown: 1) June 1st-30th; 2) July 1st-3rd		rank sum test	- CO, NO_2 and SO_2 increased during the post-lockdown period	death if infected by COVID-19
[97]	NCT Delhi, India	Assess the impact of the lockdown and meteorology on air quality	$PM_{2.5}$, PM_{10}, CO, SO_2, O_3, NH_3, NO_x, Benzene, Toluene	Daily average	T, WS, RH, AQI (calculated)	Not specified (34)	3 periods in 2020: - Pre-Lockdown: PL1: January 1st-31st PL2: February 1st-29th PL3: March 1st-23rd	Same period in 2018-2019 and comparison between periods of measurement	Spearman correlation, Principal Component Analysis, Inverse Distan	- $PM_{2.5}$ and PM_{10} decreased by 58.9% and 57.1%, respectively during the lockdown period, remaining at low values during the first phase of the lifting<br><br>- NO_x and CO also reduced about 61% and 49%, respectively	AQI improved during the lockdown

							- Lockdown: LD1: March 24th to April 14th LD2: April 15th to May 3rd LD3: May 4th-17th LD4: May 18th-31st - Post-Lockdown: UL1: June 1st-6th		ce Weighting		
[44]	America and Europe	Study the impact of the lockdown on air quality in 12 cities	SO_2, PM_{10}, NO_2, $PM_{2.5}$, CO, O_3	Daily average, daily average of the highest 8h level (for O_3)	Public transit usage, AQI (calculated)	Not Specified	January 15th to May 31st, 2020	Same period in 2019	Descriptive Statistics	-AQI improved, except in the city of Barcelona (unchangeable)<br><br>- The cities with the highest increase in the AQI were Stockholm, around 30%, for class I (good), Rome, about 15%, for class II (moderate) and Istanbul, around 25% for class III (unhealthy)	In general, public transport usage reduced between 63% and 89%, among the 12 cities studied

									- In general, the pollutants' concentrations decreased, with CO registering a significant decrease (about 92%) in Istanbul	
[117]	Naples, Italy	Study the impact of the lockdown on air quality	Benzene, CO, NO_2, SO_2, PM_{10}, $PM_{2.5}$	Daily average, average concentration	PM_{10}, $PM_{2.5}$, PM_1 (data from local OPC), aerosols properties (AERONET)	Urban background (1), suburban background (1), urban traffic (1) and industrial (1)	2 periods in 2020:<br><br>- Pre-lockdown: January 1st to March 12th<br><br>- Lockdown: March 13th to April 30th	Same period in 2019 and comparison between periods of measurement	Descriptive Statistics	- A significant reduction of NO_2, CO and SO_2 occurred in the urban traffic and industrial areas, while in the background zones little changes were registered<br><br>- PM_{10} and $PM_{2.5}$ concentrations also reduced, even though with lower extent	AOD increased during the lockdown, contrasting with the reduction of PM in urban and industrial areas
[98]	West Bengal, India	Evaluate the impact of the lockdown on air quality in Kolkata	NO_2, CO, O_3, PM_{10}, $PM_{2.5}$, SO_2, NH_3	24h average, 8h average (only for CO and O_3)	NO_2 (satellite data), AQI	Monitoring sites from KMC (6) and HMC (4)	2 periods, in 2020:<br><br>- Pre-lockdown: February 22nd to March 23rd	Comparison between the two periods of measurement	Inverse Distance Weighting, ANOVA	Overall, the O_3 concentration increased during the lockdown, which is inversely correlated to NO_2 and NH_3 that in turn decreased during the lockdown,	- Lockdown helped to reduce air pollution, enabling AQI levels to change from "poor" to

		and Howrah					- Lockdown: March 24th to May 3rd		(two-way), cluster analysis (based on Euclidean distance and Ward's methods), Pearson moment correlation coefficient	aside to $PM_{2.5}$ and PM_{10}	"good" category, in comparison with the pre-lockdown period<br><br>- The lockdown proved to be an event that helps to reduce the air pollution, since the main cause of this (human activities) were reduced
[107]	North cities, India	Evaluate the changes on air pollution due to the lockdown in Delhi,	$PM_{2.5}$, PM_{10}, NO_2, O_3, CO	Daily average and 8h average (for O_3)	T, RH, WS, planetary boundary layer height	Not specified	March 24th to April 14th 2020	Same period in 2018-2019 and comparison with pre- and during lockdown periods	Pearson's correlation	- During the lockdown, $PM_{2.5}$, PM_{10}, NO_2 and CO registered the highest reductions, among the 4 cities, respectively: 49,6%, 43%, 71.8% and 60%.	The high solar radiation and temperature and the decrease of the relative humidity may have explained

		Gurugram, Noida and Agra								On the other hand, O_3 increased drastically up to 98%<br><br>-The pollutants' concentrations reduced significantly during the lockdown, in comparison to 2018-2019	the levels registered of O_3
[81]	India	Assess the impact of the lockdown and lifting on air quality in Mumbai and Delhi	$PM_{2.5}$, PM_{10}, CO, NH_3, NO_2, SO_2, and O_3	Hourly	AQI (calculated)	Monitoring sites from Delhi (36) and from Mumbai (9)	3 periods in 2020:<br><br>- Pre-Lockdown: January to March 23rd<br><br>- Lockdown: March 24th to May 30th<br>- Post-lockdown: May 31st to June 30th	Comparison between periods of measurement and with 2018-2019 lockdown period	Descriptive Statistics	- $PM_{2.5}$ reduced from 200 μg/m^3 to 20 μg/m^3 in Delhi, and from the range 60 μg/m^3 -120 μg/m^3 to 10 μg/m^3 - 40 μg/m^3 in Mumbai during the lockdown, relatively to the pre-lockdown period<br><br>- PM_{10} reduced from 225 μg/m^3 to 60 μg/m^3 in Mumbai, NO_2 decreased from 50 μg/m^3 - 150 μg/m^3 to 20 μg/m^3 - 60 μg/m^3 in Delhi, and NH_3 decreased from 75 μg/m$_3$ to 60 μg/m^3 in Delhi during the	- National AQI changed to the "good" category, during the lockdown, changing to the "satisfactory" category in the post-lockdown period<br><br>- The lockdown did not represent an effective long-term policy to

								lockdown, in relation to the pre-lockdown period<br><br>- CO decreased from 1.5 mg/m^3 to 0.5 mg/m^3, increasing to 1 mg/m^3 during the post-lockdown period in Delhi, registering similar concentrations in Mumbai<br><br>- O_3 increased about 2.37% during the lockdown, compared to the same period in 2019	improve air quality
[64]	Pakistan	Study the impact of the lockdown on air quality, in Lahore and Karachi, using multifractal analysis	$PM_{2.5}$	Hourly	N/A	Urban background	February 1st - April 14th, 2020, with the beginning of the lockdown on March 24th	Comparison between the two periods of measurement	Multifractal time series analysis	- In Lahore, a decline of $PM_{2.5}$ was registered during the lockdown, compared to the pre-lockdown period when $PM_{2.5}$ concentration was increasing<br><br>- In Karachi, $PM_{2.5}$ reduced since the pre-lockdown period	A further multifractal analysis of PM_{10}, NO_2, NO_x, O_3 and SO_2 is needed, to fully understand the impact of the lockdown on air quality, as well as an

										annual pollutants' concentrations evaluation
[45]	Europe	Assess the impact of the lockdown on air quality in United Kingdom, France, Spain, Sweden, and Northern Italy	NO_2, PM_{10}, $PM_{2.5}$	Monthly average	NO_2, PM_{10}, $PM_{2.5}$ (satellite data), Industrial production index	Not specified	3 periods in 2020:<br><br>- Pre-Lockdown: January 1st to February 29th<br><br>- I period (lockdown): March to April<br>- II period: May	Same period in 2018-2019	Pearson's correlation, Mann-Kendall Test, Sen's Slope	- During the I period, NO_2 decreased by 31.9%, 46.8%, 41.1%, 38.2%, respectively in United Kingdom, Spain, Northern Italy, and France, while in the II period, it reduced about 41.6%, 35.4%, 31.4%, and 30.7%, relatively to 2019<br><br>- In Sweden, where a strict lockdown wasn't imposed, the NO_2 reduction was about 13.9% and 15.9% for periods I and II, respectively	- $PM_{2.5}$ and PM_{10} changes revealed to be connected with other factors beyond the lockdown<br><br>- Industrial production index proved not to be strongly correlated with the changes on air pollution
[82]	Delhi, India	Study the impact of the lockdown	PM_{10}, $PM_{2.5}$, NO_2, SO_2, O_3	Daily average	AOD (satellite data)	Not Specified	5 periods in 2020:	Same period in 2019 and between the periods of	Descriptive Statistics	- In Delhi, PM_{10}, $PM_{2.5}$, NO, NO_2 and CO concentrations decreased around 58%, 47%, 76%, 68%	- AOD reduced by 30%<br><br>- The O_3 concentrations

		on air quality					- Pre-lockdown: March 1st - 21st<br><br>- P1: March 24th-April 14th<br>- P2: April 15th-May 3rd<br>- P3: May 4th-17th<br>- P4: May 18th-31st	measurements		and 58%, respectively, during the lockdown in comparison to the reflected in 2019<br><br>- In comparison with the pre-lockdown period $PM_{2.5}$ reductions were 46%, 47%, 35% and 32%, respectively during P1, P2, P3 and P4<br><br>- O_3 increased around 4%, 45%, 97% and 88%, respectively	increase was connected to the NO and NO_2 reduction and to the high intensity of the solar radiation
[83]	Guiyang, China	Study the impact of the lockdown on air quality	$PM_{2.5}$, PM_{10}, SO_2, NO_2, CO and O_3	Average concentration	AQI, sunshine hours, T, WS, RH, Rainfall	Not specified (10)	2 periods in 2020:<br><br>- Pre-Lockdown: January 1st-22nd<br><br>- Lockdown: January 23rd to February 13th	Same period in 2017-2019	Inverse Distance Weighting	- PM_{10}, $PM_{2.5}$, NO_2, and CO reduced by 16.45%, 6.23%, 49.88% and 18.18%, respectively during the lockdown relatively to the pre-lockdown period, and decreased by 31.19%, 26.54%, 50.12% and 16.7%, respectively, compared to the previous years	AQI decreased about 7.4% and 23.48%, compared to the pre-lockdown period and to the previous years, respectively

										- SO_2 and O_3 concentrations increased about 4.6% and 31.94%, accordingly	
[68]	Wuhan City, China	Assess the effects on air quality due to the lockdown	$PM_{2.5}$, PM_{10}, SO_2, NO_2, CO, O_3	24h average, 1h	T, WS, WD, RH	City coverage (11)	3 periods in 2020:<br><br>- Pre-Lockdown: January 1st – 23rd<br><br>- Lockdown: January 24th to April 5th<br><br>- Post-Lockdown: April 6th to June 20th	Comparison between pre-, during and post-lockdown and lockdown period in 2017-2019	ANOVA, Dunn's test, Pearson's correlation	In comparison to pre-lockdown, NO_2, $PM_{2.5}$, and PM_{10} reduced about 50.6%, 41.2%, and 33.1%, respectively, whereas O_3 increased	- The increase of O_3 was possibly due to the solar radiation and temperature raise. Besides, O_3 levels were higher in 2020 in comparison to the previous years due to reduced NO_x emissions<br><br>- Right after the lockdown, the pollutants' concentrations increased, namely NO_2, O_3 and PM_{10}

[84]	Abu Dhabi, United Arab Emirates	Assess the impacts of the lockdown on air quality	NO_2, SO_2, CO, Benzene, PM_{10}, $PM_{2.5}$, O_3	Daily average, daily max 8h average (for O_3)	$PM_{2.5}/PM_{10}$ ratio (calculated), NO_2 (satellite data), mobility data, WS, T, net radiation	Urban traffic (1), Urban background (1), Suburban Background (4), and Suburban Industrial (2)	3 periods in 2020:<br><br>- Pre-Lockdown: January 1st to March 21st<br><br>- Lockdown: March 22nd to June 24th<br><br>- Post-Lockdown: June 25th to October 24th	Same period in 2019	Linear regression models	The pollutants' concentrations decreased during the lockdown, increasing in the post-lockdown period, even though remaining at lower levels than those registered in 2019	N/A
[85]	Canada	Study the changes on air quality and urban traffic volume during the lockdown in 8 cities	NO_2, CO, SO_2	Monthly average, daily average	NO_2 (satellite data), CO_2 emission (estimated), AQHI, data of motor gasoline consum	Not specified	February to August 2020	Same period in 2018-2019	Descriptive Statistics	The pollutants' concentrations decreased, with exception of SO_2 that did not register any significant change	The motor gasoline consumption and associated CO_2 emissions reduced during the lockdown in April, increasing in May

					ption, traffic index data					
[39]	World wide	Investigate the impact of the lockdown on air quality	$PM_{2.5}$, NO_2, O_3	Daily average, monthly average	N/A	Urban and/only traffic, background, industrial, semi-rural area (458)	January 1st to April 30th, 2020	Same period in 2015-2019	Signed Rank test, Paired t-test, ANOVA, Time Series Decomposition	NO_2 and O_3 had the reduction and increase globally, respectively. $PM_{2.5}$ also reduced globally	N/A
[22]	Spain	Study the lockdown repercussion on air quality in 4 cities	SO_2, CO, NO_2, PM_{10}, $PM_{2.5}$, O_3, BTXs, NH_3	Monthly average	NMHC	Urban traffic (1), suburban background (1), industrial and residential influence (1), and national coverage background	2 periods, in 2020:<br>- Pre-Lockdown: January to February<br>- Lockdown and de-escalation: March 14th to June 30th	March-June in 2013-2019 and comparison between periods of measurement	Student's t-test, Mann-Whitney U test	- NO_x, BTX_s, CO, NMHC, and NH_3' reduced statistically significant in March and April<br>- PM_{10} and $PM_{2.5}$ changes were small due to natural and residential sources	- Gradual de-escalation (since May 4th) led to a recovery of around 80% in the cities highly influenced by traffic<br>- The air quality during the period measured was better than the

										average air quality of the previous seven years (2013-2019)<br><br>- O_3 was not affected by the lockdown
[30]	Northern China	Study the impact of the lockdown on air quality, with minimization of weather and other environmental influences	$PM_{2.5}$, NO_2	Daily average	RH, WD, WS, Sea Level Pressure, planetary Boundary Layer Height	Not Specified	January to December 2020	Same period in 2015-2019	Descriptive Statistics	$PM_{2.5}$ and NO_2 decreased 0.03 μg/m^3 and 17.13 μg/m^3	The lockdown helped to ameliorate the air quality, especially by reducing the concentration of the gaseous pollutants
[55]	China	Study the impact of the lockdown on air pollution in 325	$PM_{2.5}$, PM_{10}, SO_2, NO_2, CO	Daily	AQI, population migration, T, air pressure, WS, WD,	Minimum of 1 urban site in each city	January 1st to 2nd May 2020	Comparison between periods of measurement	Simple ordinary leasts squares	The concentrations of SO_2, $PM_{2.5}$, PM_{10}, NO_2, and CO reduced by 4%, 13.1%, 15.3%, 3.3%, and 3.3%, respectively	- The avoided premature deaths due to the improvement in air quality during the lockdown, in

		cities of China			Precipitation						relation to 2018, was around 26,385 to 38,977 for China<br><br>- AQI decreased 12.2%, which statistically meant a significant reduction at 1% level<br><br>- Only a temporary reduction on air pollution was observed
[69]	China	Study the impact of the lockdown on air quality in nine cities	$PM_{2.5}$, PM_{10}, SO_2, NO_2, CO	Daily average, monthly average	COVID-19 confirmed cases	Not Specified	2 periods in 2020:<br><br>- Lockdown: January to March<br><br>- Lifting: April to September	Comparison with 2017-2019	Spearman's correlation, regression model	- PM_{10} and NO_2 registered the highest reductions, about 39% and 46%, respectively<br><br>- During the lifting period, PM_{10} and NO_2 increased by 44% and 87%	- The increase of the pollutants' concentrations during the lifting revealed to be higher than the average concentration of the

											pollutants in 2017-2019<br><br>- The air quality improved during the lockdown, but diminished during the lifting period
[86]	China	Determine the impact of the lockdown on air quality and on the heterogeneity in different urban areas, in 31 cities	$PM_{2.5}$, PM_{10}, SO_2	Average concentration	T, WS, Rainfall, Snow, COVID-19 cases	Urban	January to February, divided into 3 periods, in 2020:<br>- 10 days before the Lunar New Year<br><br>- Lunar New Year to the Lantern Festival<br><br>- 13 days after the	Same period in 2018-2019	Interrupted time-series analysis	- PM_{10} and SO_2 decreased by 15.28 μg/m^3 and 6.55 μg/m^3, respectively, relatively to 2018<br><br>- In relation to 2019, $PM_{2.5}$, PM_{10}, and SO_2 reduced 7.4 μg/m^3, 19.34 μg/m^3 and 1.41 μg/m^3, respectively<br><br>- The pollutants' concentrations reduction was statistically significant	N/A

							Lantern Festival				
[87]	China	Study the impact of the lockdown on air quality through a quasi-difference-in-difference in 367 cities	SO_2, NO_2, CO, O_3, PM_{10}, $PM_{2.5}$	Daily average	AQI, COVID-19 Infection data, meteorological data	Downtown areas	January 3rd to April 22nd, 2020	Same period in 2019	Only Descriptive Statistics	- CO and NO_2 registered the higher reductions of 30% and 20%, respectively<br>- O_3 concentration increased by 3.74%	AQI decreased about 7%, during the lockdown, rapidly increasing as soon as the number of infections by COVID-19 dropped
[31]	China	Evaluate the impact of the lockdown on air quality in 341 cities	NO_2, CO, O_3, PM_{10}, $PM_{2.5}$, SO_2	Daily average, monthly average, 1h, and 8h (only for O_3) average	AQI and Normalised Difference Vegetation Index (NDVI)	Not specified	January 1st- June 31st, 2020, with the lockdown on January 23rd to March 27th	Same period in 2014-2019 and between pre- and during the lockdown	Pearson's correlation, t-test, linear regression	- Overall, comparing pre- and during the lockdown periods, $PM_{2.5}$, PM_{10}, SO_2, CO and NO_2 reduced by 35.59%, 38.52%, 20.81%, 31.10% and 55.10%, and O_3 increased by 82.52%<br>- This behaviour was also observed when comparing the data with previous years	- AQI reduced by 39.03% during the lockdown when compared with the pre-lockdown period, and diminished by 25.83% when compared with previous years<br>- Higher NDVI (hence vegegattion

	coverage) induced a lower AQI;

AQI – Air Quality Index; LST – Land Surface Temperature; AOD – Aerosol Optical Depth; N/A – Not Applicable; WS – Wind Speed; T – Air Temperature; RH – Relative Humidity; h – Hour ; max – Maximum; SaA – Sub-area A; SaB – Sub-area B; CTRL – Reference period comparable (meteorologically) with the lockdown periods; THC – Total Hydrocarbons; NMHC – Non-Methane Hydrocarbons; WD – Wind Direction; ANOVA – Analysis of Variance; RAD – Global Solar Radiation; RMSE – Root Mean Square Error; AE – Mean Absolute Error; WHO – World Health Organization; PSI – Pollutant Standard Index; KMC – Kolkata Municipal Corporation; HMC – Howrah Municipal Corporation; WRF-AERMOD – Weather Research Forecast – Air Quality Dispersion Modelling System; MSE – Mean Square Error; MAPE – Mean Absolute Percentage Error; NAQI – National Air Quality Index; WRF-CMAQ – Weather Research Forecast – Community Multiscale Air Quality Modelling System; PVPT – Passenger Volume of Public Transportation; PVT – Passenger Volume of Taxis; AQHI – Air Quality Heath Index; ASI – Air Stagnation Index; AHF – Anthropogenic Heat Flux ; OPC – Optical Particle Counter; AERONET – Aerosol Robotic Network;
